# SPIONs Conjugate Supported Anticancer Drug Doxorubicin’s Delivery: Current Status, Challenges, and Prospects

**DOI:** 10.3390/nano12203686

**Published:** 2022-10-20

**Authors:** Naseem Akhtar, Hamdoon A. Mohammed, Mohammed Yusuf, Amal Al-Subaiyel, Ghassan M. Sulaiman, Riaz A. Khan

**Affiliations:** 1 Department of Pharmaceutics, College of Dentistry & Pharmacy, Buraydah Private Colleges, P.O. Box 31717, Buraydah 51418, Qassim, Saudi Arabia; 2 Department of Medicinal Chemistry & Pharmacognosy, College of Pharmacy, Qassim University, Buraydah 51452, Qassim, Saudi Arabia; 3 Department of Clinical Pharmacy, College of Pharmacy, Taif University, P.O. Box 11099, Taif 21944, Mecca, Saudi Arabia; 4 Department of Pharmaceutics, College of Pharmacy, Qassim University, Buraydah 51452, Qassim, Saudi Arabia; 5 Division of Biotechnology, Department of Applied Sciences, University of Technology, Baghdad 10066, Iraq

**Keywords:** superparamagnetic iron oxide nanoparticles, SPIONs, drug delivery, doxorubicin, cancer, polymer conjugation, encapsulation, dose management, controlled delivery, site specificity, drug transport, drug deloading

## Abstract

Considerable efforts have been directed towards development of nano-structured carriers to overcome the limitations of anticancer drug, doxorubicin’s, delivery to various cancer sites. The drug’s severe toxicity to cardio and hepatic systems, low therapeutic outcomes, inappropriate dose–demands, metastatic and general resistance, together with non-selectivity of the drug have led to the development of superparamagnetic iron oxide nanoparticles (SPIONs)-based drug delivery modules. Nano-scale polymeric co-encapsulation of the drug, doxorubicin, with SPIONs, the SPIONs surface end-groups’ cappings with small molecular entities, as well as structural modifications of the SPIONs’ surface-located functional end-groups, to attach the doxorubicin, have been achieved through chemical bonding by conjugation and cross-linking of natural and synthetic polymers, attachments of SPIONs made directly to the non-polymeric entities, and attachments made through mediation of molecular-spacer as well as non-spacer mediated attachments of several types of chemical entities, together with the physico-chemical bondings of the moieties, e.g., peptides, proteins, antibodies, antigens, aptamers, glycoproteins, and enzymes, etc. to the SPIONs which are capable of targeting multiple kinds of cancerous sites, have provided stable and functional SPIONs–based nano-carriers suitable for the systemic, and in vitro deliveries, together with being suitable for other biomedical/biotechnical applications. Together with the SPIONs inherent properties, and ability to respond to magnetic resonance, fluorescence-directed, dual-module, and molecular-level tumor imaging; as well as multi-modular cancer cell targeting; magnetic-field-inducible drug-elution capacity, and the SPIONs’ magnetometry-led feasibility to reach cancer action sites have made sensing, imaging, and drug and other payloads deliveries to cancerous sites for cancer treatment a viable option. Innovations in the preparation of SPIONs-based delivery modules, as biocompatible carriers; development of delivery route modalities; approaches to enhancing their drug delivery-cum-bioavailability have explicitly established the SPIONs’ versatility for oncological theranostics and imaging. The current review outlines the development of various SPIONs-based nano-carriers for targeted doxorubicin delivery to different cancer sites through multiple methods, modalities, and materials, wherein high-potential nano-structured platforms have been conceptualized, developed, and tested for, both, in vivo and in vitro conditions. The current state of the knowledge in this arena have provided definite dose-control, site-specificity, stability, transport feasibility, and effective onsite drug de-loading, however, with certain limitations, and these shortcomings have opened the field for further advancements by identifying the bottlenecks, suggestive and plausible remediation, as well as more clear directions for future development.

## 1. SPIONs: Outlook, Applications, Biological Outreach, and Limitations

**Figure 1:**
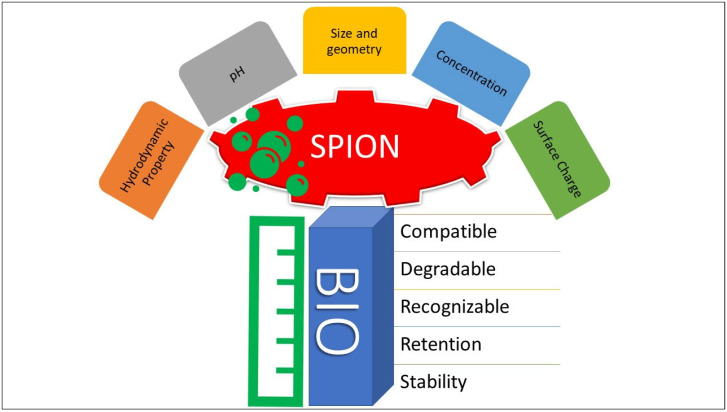
Desired characteristics and functional properties of SPIONs.

Nano-structured devices and their contributions to drug delivery have made significant progress in the diagnosis and treatment of oncological conditions. The nanometer-sized range, generally less than 100 nm, have certain unique characteristics (i.e., magnetic, electric, and optical properties) that have helped in their use in various therapeutic applications in chronic and acute physiological, and other biomedical conditions. One of the ultimate goals of biomedical research and technological advancements have been to create, and innovate new drug delivery systems at the nano-scale levels, with high payload burden efficiency, increased biocompatibility, enhanced targeting capabilities, and superior stability. Doxorubicin (DOX, also termed as Adriamycin®), the widely used anticancer agent, has encountered a number of delivery-related, physiological, and pharmacological obstacles, which have previously—and to some extent still do—restricted its widespread use as a first-line treatment choice for oncological purposes, despite having the requisite cytotoxicity. Among these hurdles, cardiotoxicity, a limited therapeutic index, the development of drug resistance, and a lack of selectivity, as well as site specificity have resulted in inadequate therapeutic outcomes, and at times total failure of treatments [1,2]. A number of developed magnetic nanoparticulate delivery vehicles have created the opportunity to address some of these limitations, and redress the hurdles in therapy for these classes of chemotherapeutic agents belonging to anthracycline category of antibiotics. These redresses were achieved through lowering the side-effects by modifying the drug to prodrug and development of DOX-conjugated entities as formulations, and certain other limitations regarding the drug stability, drug loading, systemic transport, and final deloading of the drug at the cancer site, to effectively address the therapeutic regime’s shortcomings and treatment expectations, were developed [3,4]. Superparamagnetic iron oxide nanoparticles (SPIONs) have found applications in preparation of magneto-chemotherapy (magnetic property led tumor targeting) modules, as well as in the development of other biomedical-engineering-based delivery systems. SPIONs-based delivery vehicles have also found their participation in gene transfers at subcellular levels, their capacity to overcome the chemotherapeutic failures originating from the complex incapability of the anticancer agent in structure and function, and the shortcomings in the drugs’ delivery to the intended cancer sites [5,6]. SPIONs have significantly distinct critical features (e.g., molecular size, pH, hydrodynamic properties, surface charge, concentration, colloidal properties in the medium holding them, and inherent magnetic properties at higher parametric levels), in comparison to other magnetic materials utilized for the similar purposes. Some of the desired characteristics of SPIONs include their outstanding suitability for drug delivery purposes, and their physicochemical characteristics, including size, shape, charge, and flocculation. The biodegradation properties that are dependent upon the quality of the handling biosystem, biocompatibility, and structural and morphological stability of the SPIONs in and at the required cancer sites, and that’s too for a sufficient duration of stay, along with, at times, desirable period of bioretention in the delivered system, and characteristics to cope with the fast transport and removal from the biosystem have made the SPIONs-based nano-carriers among the available state-of-the-art nanomedicine modalities (Figure 1). 

**Figure 2:**
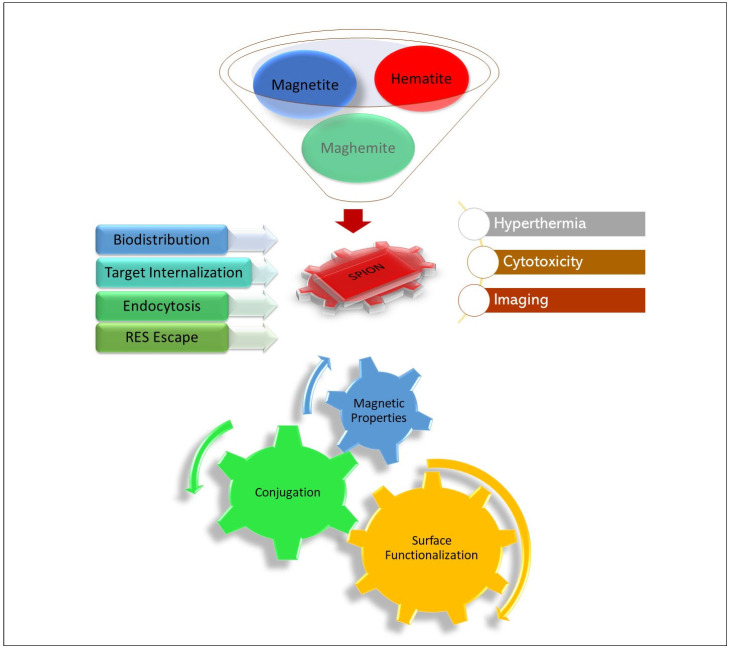
SPIONs’ core types, modification-activation, bioresponses and biological activity.

Magnetite (Fe_3_O_4_), maghemite (γ-Fe_2_O_3_), and hematite (α-Fe_2_O_3_) are the three forms of iron oxide that can make up the core of a SPION. Among all three types of SPIONs, magnetite (Fe_3_O_4_) and maghemite (γ-Fe_2_O_3_) are among the most commonly used SPIONs types. The SPIONs have been established as the choicest modality among modern oncological therapy advances for providing cures for cancer, and concurrent imaging, due to the facts of their core types’ versatility of structural, functional, and physico-chemical properties assisting in therapy, together with their functionalization that is achieved through various types of physical interactions and chemical attachments based activation strategies. The SPIONs bioresponses at the site, eventual outcomes of hyperthermia, cytotoxicity, tissue annihilation, imaging, and magnetic monitoring, have provided needed impetus to this delivery module (Figure 2). The uniquely inherent magnetic properties of SPIONs have overtly made it possible for them to be visualized by magnetic resonance imaging (MRI), magnetic-field-inducible drug-elution, magnetic navigation using fluorescence imaging, dual-module molecular tumor imaging, as well as multimodal tumor cell targeting, all of which have led to drug delivery innovations to higher levels over the last few years [7,8]. The SPIONs magnetic nanoparticles (MNPs) have well-known, and controlled interactions with externally applied magnetic fields, and thus, are considered extremely useful as drug delivery vehicles [9]. The SPIONs can also be optimized for their delivery, quantity, residence in tissue, and tracked throughout by the magnetic resonance imaging (MRI), recently developed nuclear magnetic resonance (NMR) methods, and other available processes and imaging and sensing tools. The SPIONs based delivery technique has also been successfully used as a standard theranostic model for a variety of chronic diseases other than cancers [10,11]. 

Nonetheless, the SPIONs exhibit size-dependent superparamagnetic behavior. Single domain SPIONs do not exhibit magnetization in absence of magnetic field, but show massive and combined magnetic moments, or magnetic spins in the presence of magnetic field, and orient themselves with the magnetic field. The SPIONs colloidal fluid responds to the magnetic field as a single entity. They also reduce the T1 and T2/T2* (relaxation) times in spin display. Notwithstanding, they are mostly used as a T2 contrast agent for MRI and are also available as a commercial sample, Resovist^®^, from Schering AG [12]. The eventual leaching and/or accumulation of delivered nano-scale entities, e.g., SPIONs, and the iron ions reaching to different body parts and tissues, and the SPIONs alterations in characteristics through their surface’s chemical treatments, and surface-located end-groups’ modifications have immensely contributed to homeostatic imbalance, which has eventually provided deviations in immune elements and immune-responses, together with alterations in the SPIONs bioactivities, e.g., cytotoxicity, inflammation, and DNA (deoxyribonucleic acid) damage and repairs. These outcomes have also been obtained during the treatment. Therefore, to avoid these pitfalls of the iron ions and other susceptible iron species of various genre, including nano-scale iron/iron oxide entities, a suitable chemical coating to produce surface functionalized nanoparticles (*f*-NPs) and SPIONs deemed necessary to attain the requisite properties of stability, inertness, and functional switching-on at appropriate time and situation, necessarily needed for delivery purposes, and other in vivo applications, which have been duly proposed and achieved [13,14]. SPIONs have frequently been capped/coated, encapsulated, and surface-modified through conjugation with certain chemicals, and biodegradable polymers, e.g., polyethylene glycol (PEG), poly (lactic-co-glycolic) acid (PLGA), polyethyleneimine (PEI), polyvinyl alcohol (PVA), poly (L-lysine) (PLL), starch, and dextran, etc. Such coatings are inflicted either before, or after the production of the SPIONs to avoid polymerization, flocculation, aggregation, agglomeration, biodegradation, and activity (catalytic, oxido-reductive, conjugative, etc.) losses, when administered in the in vivo and in vitro conditions [14,15,16]. The frequently used PEGylation method, other methods of surface modifications, chemical entities conjugation, and chemical attachments of single, or multi-block polymers are examples of some of the generally used procedures that can result in SPIONs’ derivatives having desired properties deemed fit for delivery and various bioapplications with their, largely to a certain extent, retained inherent capabilities, i.e., magnetic character and reactivity, that immensely help in drug and other payload deliveries [17,18,19]. Moreover, among the major approaches for preparing the SPIONs, multiple preparative methods, e.g., sol-gel, co-precipitation, polyol, thermal degradation, LASER (light amplification by stimulated emission of radiation)-assisted synthesis, electrochemical, spray pyrolysis, and hydrothermal, as well as modified double-emulsion solvent evaporation, and sonochemical techniques are common. Some of the techniques for the nanoparticles preparations are bio-inspired, bio-supported, and also biomimetic in nature. The final targeting of the nano-scale moieties have been achieved through modification of the naked SPIONs through conjugation with different polymers, co-polymers, mixed polymers, monomers, liposomes, β-cyclodextrin, small molecules, metallic entities/Au, biomolecules, i.e., peptides, proteins, glycoproteins, antibodies, antigens, aptamers, synthesized/modified sequences of different polymeric chemical classes of synthetic and natural origins, DNA, RNAs, and multiple carbohydrate variants, as well as citric and folic acids, and other small molecule entities, have also been utilized for SPIONs stabilization and modification purposes [20]. 

**Figure 3:**
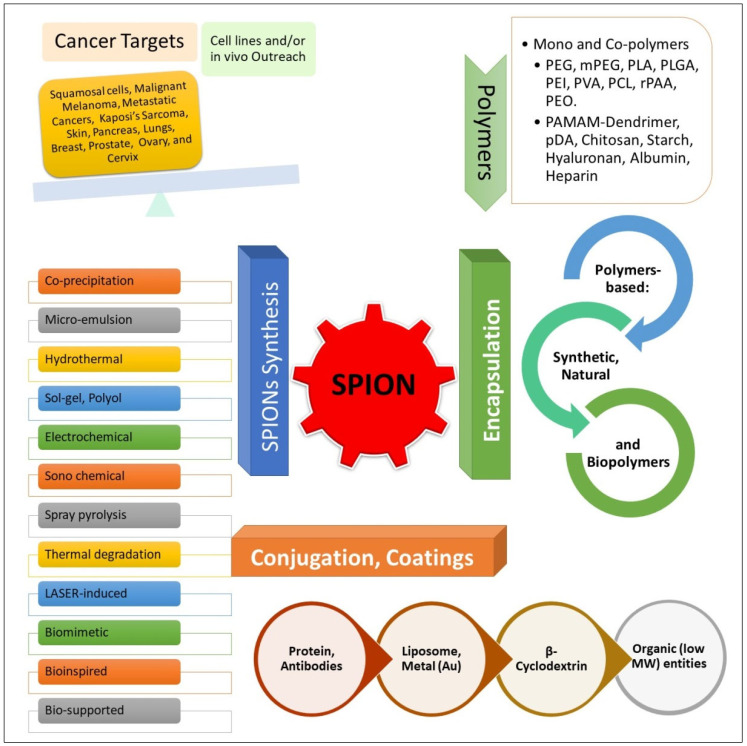
SPIONs preparative methods, encapsulation, conjugation, coatings, payloads, and targets.

Current outline provides an overview of the SPIONs up-to-date status, including achievements, drawbacks, and prospects on primarily polymer-based conjugation to the anticancer agent DOX for delivery to cancer sites, with a particular focus on the capabilities that have established this module as a high-potential nano-platform for efficient delivery of the anticancer drug DOX in oncological chemotherapy (Figure 3). 

## 2. DOX Delivery: SPIONs’ Polymeric, Monomeric, and Miscellaneous Conjugates

### 2.1. Synthetic Single-Polymer-Based DOX Conjugates

**Figure 4:**
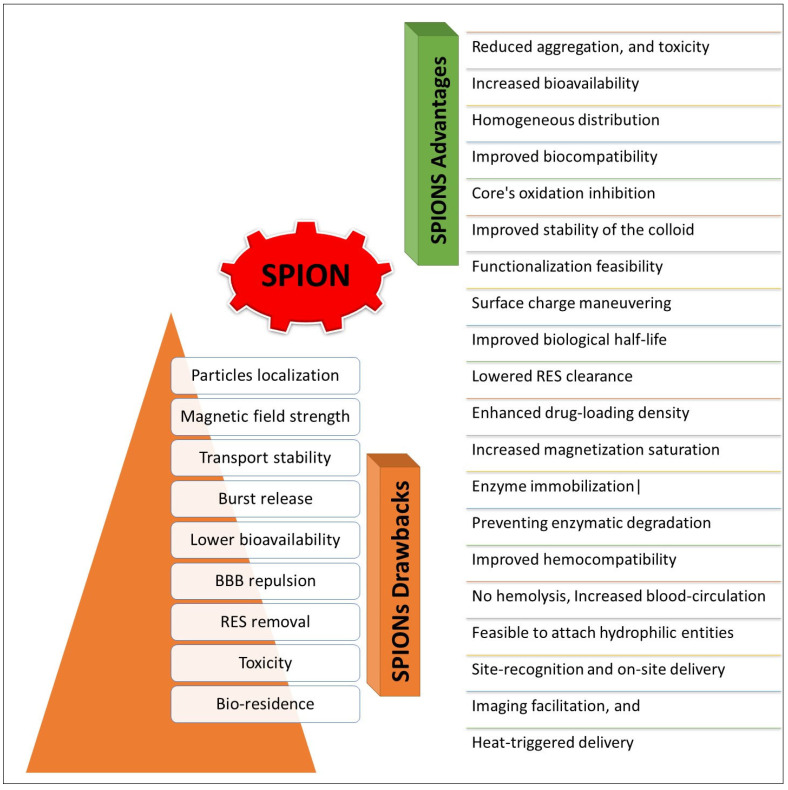
SPIONs’ advantages and shortcomings.

SPIONs produced as naked particles’ colloidal systems through various physico-chemical preparative methods for bioapplications have shown detrimental effects, and have exhibited diminished stability, poor biocompatibility, toxicity, RES (reticular endothelial system, also termed mononuclear phagocyte system (MPS) leaching and uptake, longer tissue residence, and heterogeneous distributions. These obstacles in the bioapplications of SPIONs have made it pertinent to provide certain types of coverage for the naked SPIONs to avoid the pitfalls; hence, different approaches to conjugation, coating, and polymer encapsulation have been proposed. The chemical functionalization of the SPIONs surface and the preparation of SPIONs conjugated nano-platforms, utilizing various chemical entities, especially synthetic polymers, were developed. The covering of SPIONs has provided significant benefits in terms of stability, distribution, biocompatibility, and encapsulation, as well as attachment of payloads for delivery, surface-feelers or site recognition, enough load for sustained delivery, imaging, treatment, and treatments manipulated through heat- and magnetism-triggered deliveries. The drawbacks and advantages of SPIONs are a result of their surface covering through capping, conjugation, and encapsulation. This has provided a number of distinguishable advantages that play a significant role in designing and achieving the preparation of a diverse range of delivery modules fit for different purposes under various conditions of a disease and the delivery specifications associated with these conditions have been achieved [21]. Figure 4 provides a brief summary of these observations of advantages and shortcomings. 

The polymeric materials together with their mono- and co-polymeric entities, have been utilized for in vitro cancer cell lines and in vivo cancer sites drug delivery with the aims of providing stability, transport feasibility, efficient drug targeting, drug deloading at the site, and removal/excretion of residual products, metabolites, and byproducts [22].

#### 2.1.1. Polyethylene Glycol

For drug delivery systems, polyethylene glycol (PEG) is among the most prominent biodegradable and biocompatible of polymers. This US FDA (United States Food and Drug Administration) approved polymer is widely accepted owing to its biologically and chemically adaptable characteristics towards developing delivery systems, mediating safe bioaction properties as part of the developed nano-carrier module. The established safety profile and biodegradation characteristics, both of which are important factors in selecting a polymer as part of the formulation strategy, have been achieved [23]. The PEGylation (chemical attachment of PEG), physical attachment of PEGs (derivatized, small MW) as a non-covalent appendage, co-conjugation with other (non-polymeric) entities, and surface modification using PEG-derived polymers with SPIONs are among the most explored approaches for preparing competent delivery modules aimed at improving the delivery of drugs and other payloads to site through the efficient targeting of cancer cells and other tissue sites in the plan of delivery [24,25]. The functions of the PEG’s polymeric length and molecular mass are the determining factors for ascertaining the suitability of polymer types for employment in drug delivery, and the inbound treatment. Surface functionalization, such as, with part of the PEG coating, has been determined to be better with phospholipid–PEG2000, which possesses a high drug loading capacity at 30.8±2.2% *w/w* (weight-by-weight). Phospholipid–PEG-coated SPIONs have been shown to be capable of elevating the local body temperature to apoptotic levels, whereby the release of DOX for an extended period without influencing the medication’s effects has been observed. SPIONs’ multimodal hyperthermia–DOX therapy was found to be more successful in triggering cell death than other comparable treatment modalities that employed the hyperthermia route [26]. The superior drug absorption, and hemo-compatibility of the developed PEGylated silica-coated SPIONs for multipurpose stimuli–responsive co-delivery of DOX and cisplatin—another chloro-platinate-based anticancer agent—have been recorded. The developed nano-formulation exhibited high photothermal properties of LASER irradiation at 0.5 w/cm^2^ with an 808 nm wavelength. In addition, a maximal cytotoxicity at 97.3±0.8% was achieved after intracellular delivery for over 10 min of LASER irradiation [27]. In another trial, the conjugation of DOX and PEG with antibodies (antiIL4R blocking) was utilized to determine the effectiveness of the optimized formulation, which provided a substantial decrease in cell viability, and led to promoting apoptosis in 4T1 cells. When compared with either DOX–SPIONs–IL4R or free-DOX alone, a combined therapy with SPIONs–IL4R loaded with DOX was found to induce substantial increments in the death, apoptosis, and oxidative stress of the cells at the cancer site, thereby showing that the combination has improved the therapeutic effects of DOX [28]. Yet, in another experiment, multifunctional, pH-sensitive, and small-sized SPIONs coupled with DOX, together with biocompatible PEG polymers, were developed, and trial experiments conducted for the treatment and imaging of tumors, through tumor magnetic resonance imaging, substantiated the feasibility of the product and the procedure. It also showed the biocompatibility, and eventually, the biodegradation of the preparation, the major constituent being the PEG. Under magnetic fields, the SPIONs preparations were observed to exhibit higher anticancer impacts than the non-PEG-bound and free-DOX formulations. These SPIONs–DOX-conjugated nano-products demonstrated significant aqueous solubility with the solution’s increased stability, as well as strong magnetizations in acidic environments. This was, apparently, possible because the breakage of the acyl hydrazone pH-sensitive linkages contained in the preparation was activated. The DOX-conjugated SPIONs nano-product demonstrated substantial cellular uptake, which further confirmed their strong targeting capabilities in the magnetic field [29]. Interestingly, in yet another investigation, magnetic SPIONs were effectively used for MRI purpose as the contrast agent. Superparamagnetic Fe_3_O_4_-based SPIONs cores with high T2 relaxivity were also formulated. The preparation was composed of PEG, DOX, protein (i.e., bovine serum albumin, BSA), and their layers were cross-linked. The DOX molecules were shown to be absorbed within the nano-preparation, probably due to the presence of hydrophobic and electrostatic interactions, together with the carbodiimide grafting of the PEG. The drug loading efficiency was at 8% *w/w*. The DOX-loaded preparations were found to rapidly release DOX at pH 7.4, and also exhibited comparable cytotoxicity against the HEK293 and C6 cell lines, as did the free-DOX [30]. Water-soluble multifunctional nano-carriers from SPIONs and DOX were developed, which were conjugated at the distal ends of the PEG arms. PET (positron emission tomography) led ^64^Cu chelators, tumor-targeting ligands (i.e., cyclo-(Arg-Gly-Asp-d-Phe-Cys), or [c(RGDFC)] peptides), and macrocyclic 1,4,7-triazacyclononane-N,N’,N”tri-acetic acid (NOTA) were coupled, and under in vitro conditions, the cyclic-Arg-Gly-Asp (cRGD)-conjugated SPIONs exhibited higher cellular absorptions and demonstrated substantially higher levels of tumor growth inhibition in comparison to the cRGD-free formulations, a feat achieved through the use of chelators, ligands, and PET imaging. SPIONs carriers also showed higher levels of efficiency in the combination therapy when targeted as part of the chemotherapeutic delivery of DOX, and they were monitored through the PET/MRI dual-modality tumor imaging technique [31]. The SPIONs-based nano-theranostics using antibodies and fluorescent labels was also employed for MRI and fluorescence imaging purposes with tumor cells, together providing intracellular drug release in a pH-dependent manner. The PEGylated SPIONs (SPIONs of 10 nm in size) were coated with an amphiphilic polymer and fluorescent dye, i.e., HuCC_49_CH_2_ and 5-FAM (5-carboxyfluorescein), respectively, together with an antibody attached to the PEG of the SPIONs. Azido-doxorubicin (A-DOX), MDM2 (mouse double minute 2 homolog, also known as E3 ubiquitin-protein ligase)-inhibitor (MI-219), and Hsp90 inhibitor (17-DMAG, 17-dimethylaminoethylamino-17-demethoxygeldanamycin, also known as alvespimycin) were incorporated into the SPIONs. DOX, A-DOX, MI-219, and 17-DMAG, with achieved loading capabilities at 3.16 ± 0.77%, 6.04 ± 0.61%, 2.22 ± 0.42%, and 0.09 ± 0.07%, respectively. Following the PEGylation, the particle sizes of the SPIONs increased to 44 nm. The antibody coupled together with fluorescent dye, 5-FAM, were conjugated and applied to track the SPIONs’ intracellular localization, while the drug’s release was observed in a pH-dependent manner. The HuCC_49_CH_2_-SPIONs enhanced the tumor cell targeting, as evidenced by the fluorescent imaging, MRI, and Prussian blue staining. The T(2) (relaxation time) values in the MRI of the LS174T cells were also reduced from 117.3 ± 1.8 ms (micro seconds) to 55.5 ± 2.6 ms when the HuCC_49_CH_2_-SPIONs nano-theranostic modality was used. Furthermore, a pH-dependent release was observed for DOX, MI-219, and 17-DMAG but not for A-DOX, wherein fluorescent imaging revealed that the HuCC_49_CH_2_-SPIONs nano-theranostic agent had accumulated in the endosomes, and cellular lysosomes. The HuCC_49_CH_2_-SPIONs nano-theranostic released higher quantities of DOX to LS174T cells and showed lower IC_50_ (1.44 *versus* 0.44 M) values than the non-specific IgG (immunoglobulin G)-SPIONs. The HuCC_49_CH_2_-SPIONs nano-theranostic platform also performed cancer cell imaging, targeted delivery of the drug, and drug delivery with a pH responsiveness [32].

#### 2.1.2. Poly-Lactic-co-Glycolic Acid

US FDA-approved poly(lactic-co-glycolic acid) (PLGA) is an important biodegradable polymer with application in drug delivery owing to its better biocompatibility with cellular/subcellular components, inherent capabilities for the controlled as well as sustained release of payloads, and minimal toxicity manifestations [33]. PLGA is one of the most commonly used polymers in the preparation of reversible carriers, with significant biocompatible and biodegradable properties and an inherent ability to control physicochemical properties through the conjugation of various structural entities both polymeric and non-polymeric in nature; chemical modification and blending; MW alterations; structural variations in the constituent lactic and glycolic acids as well as their ratio in the prepared PLGA units. The tailorable drug release parameters coupled with the unhindered rendering of the biological activity of the PLGA-based nano-structured modules holding the drug have made it the polymer of choice for the development of nano-platforms for the various requirements of delivery [34]. Gao et al. developed a multifunctional drug-loaded nano-system composed of PLGA-folic acid and DOX as SPIONs-controlled entities to reduce the toxicity and improve the diagnosis and treatment of cancers. The developed SPIONs have displayed noteworthy targeting capabilities due to the fact of the surface modifications achieved with folic acid and the actable cell-penetrating peptide (ACPP), which lowered the toxicity to the normal organs [35]. Surfactant-coated PLGA polymer loaded with SPIONs and encapsulating DOX were prepared with efficient drug loading characteristics when the PLGA’s molecular weight (MW) was increased [36]. Moreover, in another experiment, the surfactant-coated formulations were more stable at 4 °C, and the relaxivity was of a very high quality. The theranostic PLGA-based nano-formulations were also effective for in vitro therapy of glioma cells and demonstrated effectiveness as a contrast agent for MRI purposes. The investigation also discovered effective theranostic hybrid nano-formulations that can be used to deliver hydrophobic chemotherapeutic agents through MRI monitoring [37]. Yet, in another study, the authors compared four different PLGA formulations developed through the double-emulsion solvent evaporation technique, providing PLGA co-encapsulation of the SPIONs and DOX, with better payload availability, enhanced particle size, and better particle size distributions. The optimal phase ratio of the prepared SPIONs-DOX-PLGA formulation was found to be a stable delivery system for cancer therapeutics and for diagnostic purposes [34]. The existing methods for detecting malignant cells in sentinel lymph nodes, as well as towards treatment of metastases related deficiencies are some of the known drawbacks in cancer management. Therefore, PLGA-based microbubbles were used to encapsulate the SPIONs, and the chemotherapeutic drug, DOX, was formulated as part of the multifunctional polymer microbubbles (MPMBs) using perfluorocarbon gas. The developed MPMBs had small size distributions and a flat surface with a diameter of 868.0 ± 68.73 nm. In vitro experiments showed that the MPMBs could improve both ultrasound (US) and MR imaging (MRI), while the in vivo results showed that these MPMBs improved the tumor lymph nodes’ signals. Among the other indicators, tumor growth index, micro-blood vessel density, and micro-lymphatic vessel density were also assessed in the in vivo antitumor efficacy experiments of the MPMB-mediated chemotherapy. It was concluded that a DOX-loaded SPIONs PLGA-iron oxide MPMB was suitable for dual-mode US/MRI image analyses of lymph nodes together with low-frequency US-triggered therapeutic material for the intended tumor size. It offers a method for imaging and treating malignant tumors and their metastatic conditions [38]. In this work, SPIONs-Fe_3_O_4_-loaded DOX was incorporated into temperature-responsive PLGA nano-matrices to create magnetic-field-inducible drug-eluting nano-particulate (MIDEN) material for cancer treatment. An external alternating magnetic field (AMF) generated heat above 42 °C, which caused the PLGA polymer matrix (Tg = 42–45 °C) to transition from a glassy to a rubbery state and allowed for the slow release of the encapsulated DOX. Eventually, concurrent hyperthermia and local heat-triggered chemotherapy for the efficient treatment of cancer was successfully achieved. The synthesized MIDENs had a diameter of 172.1 ± 3.20 nm. In vitro investigations revealed that the MIDENs were cyto-compatible and efficient at eliminating the CT26 colon tumor cells, when used with the AMF. The MIDENs offered increased T2 contrast magnetic resonance and a substantial in vivo suppression of the malignant tumor growth under AMF. The optimized multifunctional MIDENs, which were comprised of biocompatible materials, and the employed therapeutic approaches were extremely useful in image-guided heat transfer therapy for cancer [39]. Similarly, Dong et al. developed a formulation containing SPIONs, DOX, and Her2 GPDH (human epidermal growth factor receptor 2 glycine-aspartic acid-proline-histidine) as a theranostic agent, which was composed of perfluorooctyl bromide for bimodal US/MRI purposes and provided synergistic photodynamic therapy for Her2-positive cancer cells. According to targeting assays, the Her2-GPDH nano-carriers had fine spherical structures with a diameter of 296 nm and possessed a greater target binding to Her2-positive SKBR3 cells than the Her2-negative MDA-MB-231 cells. The DOX entrapment efficiency and loading contents were high. The DOX displayed considerable photothermal cytotoxicity on the SKBR3 cells. According to the study, due to the combination therapy consisting of photothermal applications in conjunction with chemotherapy, the antitumor impacts significantly improved [40].

### 2.2. Combination/Copolymers-Based DOX-SPIONs Conjugates

#### 2.2.1. Polyethylene Glycol-Poly-Lactic-co-Glycolic Acid

Polymeric PEG material possesses distinctive hydrophilic properties, and PEGylation is widely used for enhancing water solubility for the targeted delivery of drugs and for diagnostic improvements in cancer cells [24,41]. The synergistic effects were observed when PEG was conjugated with PLGA in delivery experiments involving chemotherapeutic agents. A redox-sensitive, folate-conjugated, multi-block polymeric system (PLGA-PEG-PLGA-urethane-*ss*), as a nano-carrier module for enhancing treatment’s efficacy in cancer cells through the combined delivery of SPIONs and DOX was prepared. The developed polymeric nano-preparation was enclosed with DOX and crystalline SPIONs, with an average particle size of 170 nm and a zeta potential indicating the dispersive stability of the preparation at −33 mV, which allowed for the co-delivery of both to the cancer cells. In presence of 10 mM glutathione (GSH), the nano-carriers’ redox sensitivity was demonstrated by a 4.47-folds increase in the drug’s release. The in vitro cell line experiments after exposure of the cells to a radio frequency (RF) also produced hyperthermia at 43 °C. The optimized nano-carriers revealed combined toxic effects in folate-overexpressing cells (i.e., HeLa and MDA-MB-231), and the efficiency of the prepared nano-system was demonstrated. The system has a facile preparative process, repeatability, and the ability to deliver medication together with SPIONs to cancer cells in a combined and targeted manner. This strategy can also aid in attaining greater therapeutic benefits while minimizing the toxic effects [42]. The PEG–PLGA copolymer was chosen as a standard copolymer for the development of a nano-sized drug carrier, and the delivery system was impacted by the module’s structures, such as, NPs (nanoparticles), micelles, and hydrogels formats [43]. The magnetic characteristics of the SPIONs assisted in keeping the delivery in place at the site when an external magnetic field was applied. The entrapped DOX was at the lower quantitative scales for the PLGA:PEG (2000) and PLGA:PEG (3000) triblock copolymers but was found to significantly increase for the PLGA:PEG (4000) copolymer-based formulation. This was owing to the blended triblock copolymer’s improved water absorption ability, which entrapped more DOX molecules in the swelling stage of the copolymer matrix [44].

#### 2.2.2. Polyethylene Glycol-3-Aminopropyl Triethoxy Silane

An important surface modification process, also applicable to SPIONs, involved the use of the coupling agent, 3-aminopropyl triethoxy silane (APTES), an organo-silane molecule, which rendered a strong network for the entrapment of complex molecules [45]. PEG and APTES, when conjugated with indium-111 (^111^In)-labeled DOX, and another anticancer agent, trastuzumab (Trastu), for tumor treatments, delivery, targeting, controlled drug release, and dual-modal tumor imaging, proved to be highly effective. The SPIONs were functionalized using N-hydroxy-succinimide (NHS) ester of the PEG-maleimide (NHS-PEG-Mal) to conjugate the thiolated-3,6,9,15-tetraazabicyclo-[9.3.1]-pentadeca-1(15),11,13-triene-3,6,9,-triacetic acid (PCTA), the bifunctional chelator (BFC), and Trastu-DOX molecules, when connected to SPIONs through pH-sensitive hydrazone bonds, resulted in highly regulated drug release at the tumor site [46]. In another study, SPIONs were produced through the low-temperature co-precipitation method using two energy sources: thermal and microwaves. The thermal and microwave samples, respectively, yielded a 59% and 39% drug release at pH 5. The in vitro tests supported the applicability of the SPIONs as effective and targetable drug carriers through successful internalizations and subsequent cell apoptosis, despite the lower drug release from the microwave sample. The relevance of energy in regulating the efficiency and functional behavior of SPIONs as a nano-scale drug carrier was, again, established for their efficacy, biocompatibility, and magnetization-based performance for effective and targeted drug delivery option [47].

#### 2.2.3. Polyethylene Glycol-Poly-ε-Caprolactone

Over the last few decades, several approaches have been proposed to use the PEG-PCL co-polymer for nano-scale delivery [48,49]. The co-delivery of SPIONs and DOX as magnetic nano-micelles with a 32% loading of reduction-responsive biodegradable micelles was created by coupling the PEG and poly-ε-caprolactone with a disulfide bond (PEG-SS-PCL). Due to the disulfide linkage across the main chain, the amphiphilic diblock copolymer exhibited redox-responsive characteristics, while the hydrophobic and hydrophilic ratios of each block of polymers were uniformly selected, and the single oleic acid was linked to the Fe_3_O_4_, the magnetic component of the nano-preparation. The molecular dynamics simulation approach-led experimentation ensured the construction of self-assembled PEG-PCL micelles in the presence of oleic acid and water as a solvent. The findings revealed the self-assembled nano-micelles’ stability, cyto-compatibility, magnetic and redox-responsive abilities, and the study also suggested that the DOX-SPIONs-loaded, reduction-sensitive nano-micelles can be employed in the targeting of drugs to tumor cells [50].

#### 2.2.4. DSPE-Polyethylene Glycol 2000

DSPE-PEG (2000), a 1,2-distearoyl-*sn*-glycero-3-phosphoethanolamine-polyethylene glycol derivative and a lipidic material with a PEG chain grafted onto its hydrophobic tail, has been employed in a variety of nano-platforms, including solid-lipid NPs, liposomes and polymeric micelles preparations to improve the delivery of drugs, better cellular targeting, and drug absorption [51]. In this context, the delivery of NPs has advanced to the point that it may be able to solve the challenges faced by the thermal breakdown and to produce hydrophobic SPIONs. The chemotherapeutic agent (i.e., DOX) as well as other anticancer agents exhibit a poor blood–brain barrier (BBB) permeability, which limits their usage. Conjugation with 1,2-distearoyl-sn-glycero-3-phosphoethanolamine-N-[methoxy-(polyethylene glycol)-2000] (DSPE-PEG 2000), and in this case, the DOX loaded together with indocyanine green (ICG) into the phospholipid layers using a thin-film hydration process, offered an impressive alternative. The multifunctional SPIONs thus produced had a size of 22.9 nm and a zeta potential of −38.19 mV. In vitro tests revealed that the SPIONs coated with DSPE-PEG-DOX-ICG effectively increased DOX’s cellular transport, compared to the free-DOX alone. The nano-preparation not only crossed the BBB effectively but also preferentially accumulated at the tumor location, as observed in the in vivo fluorescent and MR imaging results. Furthermore, the C6 glioma-bearing rats treated with these SPIONs had the highest therapeutic efficacy, resulting in the lowest tumor volume, lowest body weight loss, and highest life expectancy with no noticeable adverse effects [52]. 

#### 2.2.5. Polyethylene Glycol-Polyethylene Imine

The novel co-polymer consisted of polyethylene glycol (PEG) and cationic polyethylene imine (PEI) has been well studied as part of a combination polymer that significantly increased the drug’s delivery at cellular levels [53]. An improved polyol technique was used to prepare monodispersed SPIONs co-coated with the PEG and PEI polymers. Folic acid (FA) was attached to the SPIONs’ surface using an EDC/NHS (1-ethyl-3-(3-dimethyl amino) propyl carbodiimide hydrochloride/N-hydroxy succinimide) coupling technique to provide cancer-specific targeting characteristics to FA-SPIONs. The anticancer drug DOX was loaded onto the FA-SPIONs as DOX-FA-SPIONs, and the DOX release rate from the DOX-FA-SPIONs were substantially higher in a lower pH in the PBS (phosphate buffer)-based simulation fluids. In PBS, the SPIONs, FA-SPIONs, and DOX-FA-SPIONs showed high colloidal stability, with mean hydrodynamic sizes of 23, 40, and 67 nm, respectively, of the final nano-preparations. The results of the CLSM (confocal LASER scanning microscopy) suggested that the DOX-FA-SPIONs efficiently targeted the MCF-7 cells through FA receptor-mediated endocytosis. The DOX-FA-SPIONs were examined in nude mice with xenograft MCF-7 breast cancer cells through tail intravenous injection, and were shown to be more effective at inhibiting tumor development. The DOX-FA-SPIONs’ growth suppressing efficiency on MCF-7 cancer cells in vitro and on xenograft MCF-7 breast tumor model of nude mice in vivo was considerably enhanced under a magnetic field (MF). The MRI was used to track the accumulation of SPIONs in the cancer cells as the DOX-FA-SPIONs had a high r2 relaxivity (81.77 mM^−1^S^−1^). The histology of the liver, lungs, kidneys, and heart of the mice revealed no substantial toxicity of DOX-FA-SPIONs on these and other organs of the animals after 35 days of therapy. The FA-SPIONs were found to be an effective nano-platform for drug delivery with a high efficacy for use as an advanced cancer theranostic formulation [54]. 

#### 2.2.6. Polyethylene Glycol-Block-Poly-ε-Caprolactone

Polyethylene glycol-block-poly-ε-caprolactone (PEG-PCL) co-polymers are characterized by their outstanding biodegradable properties. An anti-EGFR (epidermal growth factor receptor) monoclonal antibody was coupled to poly (ethylene glycol)-block-poly-ε-caprolactone, PEG-PCL, loaded with DOX and SPIONs. The ability of the produced immunomicelles to carry SPIONs into tumor cells using 1.5 T magnetic field, monitored through a clinical magnetic resonance imaging scanner, was found to be efficient. The observed MRI T2 signal intensity of the A431 cells treated with SPIONs-loaded and antibody-functionalized micelles were shown to drastically reduce the tumor condition. The immunomicelles also reduced the cell proliferation more efficiently than their non-targeting non-antibody functionalized counterparts, as per the thiazolyl blue tetrazolium bromide (MTT) color tests. These results implied that the immunomicelles used to deliver DOX through the SPIONs to the EGFR-overexpressing tumor cells, in the in vitro conditions, can be used as an MRI agent to visualize, and target the drug for delivery purposes for improved tumor imaging and therapy [55].

#### 2.2.7. Polyamidoamine-Polyethylene Glycol-Dodecyl Amine

The surface modification of amine-terminated polyamidoamine (PAMAM) dendrimers through polyethylene glycol groups generally enhances the water-solubility and overall biocompatibility of a drug delivery platform demanding hydrophilic conditions [56]. A self-assembled reducible co-polymer, including SPIONs that transport DOX for chemotherapy, was developed. Michael addition reaction was used to develop a co-polymer of the reducible polyamidoamine (rPAA) and PEG-dodecyl amine graft. The alkyl group grafts of the reducible co-polymers were intercalated with the oleic acid layer on the surface of the magnetite nano-crystals to generate the rPAA-SPIONs. The intercalating regions served as a reservoir for the hydrophobic DOX, while the PEG component in the co-polymer assisted in making the nano-preparation well dispersible in water. The two-photon excited fluorescence (TPEF) and coherent anti-Stokes Raman (CARS) techniques were employed to investigate the intracellular substructures in the living cells, and Vivaview^®^ technology was used to reveal the real-time inhibitory efficiency of the nan-preparation in the live cells. Moreover, the rPAA-SPIONs were also evaluated in mice with xenograft MDA-MB-231 breast tumor cell lines by i.v. (intravenous) injection, and they were shown to effectively limit the tumor’s development [57]. In another system, SPIONs were investigated for intracellular delivery, composed of a co-polymer of reducible polyamidoamine (rPAA) with PEG-dodecyl amine. The Vivaview^®^ technique showed real-time inhibition efficiency of the NPs in the live cells. The rPAA-SPIONs represented efficient drug loading, and showed favorable bioactions in the in vitro tests. Moreover, the tumor growth was efficiently inhibited in the mice though i.v. injections of rPAA-SPIONs nano-preparation. Other tests were also indicative of the DOX-rPAA-SPIONs’ reduced toxicity in the normal organs of the mice after 24 days of treatment [57].

#### 2.2.8. Polylactic Acid-Polyethylene Glycol

Polylactic acid–polyethylene glycol (PLA-PEG) block co-polymer, as well as its end-group derivative-based nano-particulate materials have been the focus of research in recent years. The co-polymer has demonstrated improvements in the uploading of hydrophobic drugs, and showed reduction in the drugs’ burst effect. It also avoided engulfment of nano-systems by the both kinds of phagocytes, i.e., professional (e.g., monocytes, macrophages, neutrophils, tissue dendritic cells, and mast cells), and non-professional phagocytes (i.e., epithelial and endothelial cells, fibroblasts, and mesenchymal cells). It has also helped to increase the circulation time of drug in the blood and has improved their bioavailability [58]. Combination therapies that used a targeted polymer and a magnetic hyperthermia approach with the chemotherapeutic non-cancer drug, DOX, have been described as a promising treatment option. The combination therapy, a cornerstone of cancer therapy, is considered an innovative cancer treatment modality. The double-emulsion approach was used to develop DOX-loaded PLA-PEG-FA magnetic SPIONs as a nano-carrier. The produced SPIONs were spherical in shape, and had little aggregation with significant magnetic characteristics. At the highest applied magnetic field strength, the saturation magnetizations of the SPIONs were at 59/447 and 28/224 emu/g, respectively, for the nano-preparations incorporating FA and without it. A hyperthermia device was used to test the heat-generating capacity of the magnetic nano-carriers in an external AC (alternating current) magnetic field. The highest temperature, 44.2 °C, was observed in the nano-carriers suspension at a *w/w* ratio of 10:1 (polymer:DOX) after 60 min of exposure to the magnetic field. The results showed that the DOX-loaded-PLA–PEG–FA-SPIONs (targeted nano-carrier incorporating FA) had a higher cellular uptake than the DOX-loaded-PLA–PEG-SPIONs (non-targeted), and the free-DOX alone. Following from this, the DOX-loaded-PLA–PEG–FA-SPIONs showed a higher rate of apoptosis than the DOX-loaded-PLA–PEG-SPIONs (non-targeted), and the free-DOX, wherein the percentage of apoptotic cells reached at 57.75 ± 3.8% and 61.81± 4.1% for the HeLa and CT26 cell lines, respectively, after 24 h (hours) of treatment with these targeted nano-carriers. As a result, the DOX-loaded-PLA–PEG–FA-SPIONs were established as a multifunctional tumor-targeting delivery system for cancer cells through an approach involving the generation of hyperthermia [59]. 

#### 2.2.9. Bis-[(*p*-sulfonato-phenyl)-phenylphosphine]-methoxy-polyethylene glycol-thiol 

Iron oxide (i.e., Fe_2_O_3_-maghemite) and SPIONs cargoes were encapsulated within the matrices of bis-((*p*-sulfonatophenyl)-phenylphosphine)-methoxy-polyethylene-glycol-thiol polymer vesicles for active targeting of anticancer drugs using a two-step process. The approach allowed for easy access to the highly reactive surface groups found on the SPIONs as well as control over the produced NPs’ diameter (50–100 nm). It was possible to load DOX and release it in cancer cells under in vitro conditions. For the instances of encapsulated DOX molecules, the possibility of controlled drug release under various pH conditions were also proven, and the validity of stimulated drug delivery for magneto-chemotherapy was again safe vouched through this approach. The increased contrast qualities of these polymer-magnetic nano-cargoes (PMNCs) also offered enhanced applications for magnetic resonance imaging [60].

#### 2.2.10. Polyethylene Oxide-Trimellitic Anhydride Chloride-Folate

Trimellitic anhydride (TMA), an important acid anhydride, is extensively used in industrial applications [61,62]. A unique polymeric nano-system comprising DOX, polyethylene oxide-trimellitic anhydride chloride-folate (PEO-TMA-FA), and SPIONs (Fe_3_O_4_) was conceptualized and prepared. The formulated SPIONs possessed increased efficiency and showed reduced toxicity. The anticancer efficacy of the preparation was assessed by comparing the NPs’ biological activity against the free-DOX, and with a commercial DOX, DOXIL^®^. The relative tumor growth in the DOX-loaded-NPs-treated group decreased by two-folds, and four-folds in the rats and rabbits models, respectively, as compared to the free-DOX alone, and DOXIL^®^-treated animal groups. Moreover, despite the lower iron contents, the DOX-loaded-NPs-treated group comparatively had higher MRI sensitivity to that observed in the typical MRI contrast agent, Resovist^®^-based experiments. The DOX-loaded-NPs-treated group showed lower activation of CD34 and Ki-67, which are biomarkers of angiogenesis and the cell proliferation, respectively, in the terminal deoxynucleotidyl transferase dUTP (deoxy uridine triphosphate) nick-end-labeling (TUNEL) assay [63].

### 2.3. Single Characteristically Specified Polymer-Based SPIONs Conjugates

#### 2.3.1. Polyvinyl Alcohol: The Thermoplastic Polymer

Polyvinyl alcohol (PVA), a semi-crystalline thermoplastic polymer, has long emerged as a promising biodegradable material for the fabrication of hydrogels, microparticles, NPs, and nano-composites for drug delivery purposes, owing to their exceptionally biocompatible and non-toxic characteristics. The characteristic was also retained when the polymer was combined with certain other polymers to give the requisite co-polymer [64]. The SPIONs in nano-shells, and a PVA-based chemoembolization system were designed for the delivery of DOX. The viability and safety of transporting the DOX and SPIONs were tested. In a rabbit liver tumor model, the SPIONs-DOX-PVA combination was delivered by a catheter into the hepatic arteries, which dramatically reduced the tumor development rate, as compared to other treatment groups, and it resulted in smaller tumor volumes and longer survival times for the experimental animals. The pharmacokinetics investigations and histological analyses of the SPIONs-DOX-PVA treated groups showed long-term retention and enhanced drug release inside the tumor. It also resulted in significantly enhanced tumor necrosis and apoptosis [65]. The impact of the coating’s concentrations of the hydrophilic polymer PVA and SPIONs-loaded-DOX on the saturation magnetization was also studied. For the purpose, the nano-preparation was used under physical immobilization, and the externally controlled movement of SPIONs in blood circulation at saturation magnetization under the modification impact of an applied magnetic fields was observed, wherein a suitable ratio of PVP weight was discovered to be at 3% *w/w* for the best working preparation [66].

#### 2.3.2. Polyethylene Imine: The Cationic Polymer

Polyethylene Imine (PEI) is among the most promising cationic carriers for polymeric micelle-based systems for drug delivery [67]. The developed DOX-MMSN-SS-PEI-SPIONs (DOX-conjugated monodisperse mesoporous silica-coated SPIONs) instantly destroyed tumor cells due to the fast release of DOX from the polymeric nano-micelles. These unique multifunctional nano-platforms successfully combined the MRI applications, feasibility of targeted drug delivery to the site, and, overall, the controlled release of the drug, which resulted in significant improvements in tumor diagnosis and therapy. Surface modification of the monodispersed mesoporous silica NPs with the polymer, polyethylene imine (PEI), through disulfide linkages by coupling of the citraconic anhydride to the PEI, comprised the majority of the manufacturing processes [68].

#### 2.3.3. Polydopamine: Amines and Catechol-Ends Polymer

Polydopamine (pDA) is another versatile polymer that has been used to build different NP-based modules as drug carriers through the surface modification approach [69]. Iron oxide core–shell NPs (SPIONs) doped with polydopamine (pDA) have been produced, and used as nano-carriers for drug delivery. The pDA-reactive quinone improved the binding efficiency of a variety of biomolecules for targeted delivery and later the drugs’ distributions. The surfaces of the pDA-SPIONs were immobilized with glutathione disulfide (GS-SG), a common thiol molecule found in the cytoplasm. The GS-SG acts as a cellular trigger, causing the medication to be released from the NPs, thus resulting in effective drug delivery. The GS-SG was also modified on the surface to create S-nitroso-glutathione, which operated as a NO (nitrous oxide) donor. The TG (thermogravimetric) analysis revealed that the anticancer drugs, DOX and docetaxel (DTX), were loaded onto the NPs with loading efficiencies of 243 and 223 mol/g on the SPIONs, respectively. In a DOX release experiment utilizing UV-Vis spectroscopy, the prepared nano-carriers showed a pH sensitive behavior, indicating that it can be employed as a drug delivery preparation. The prepared 3-(4,5-dimethylthiazol-2-yl)-5-(3-carboxymethoxyphenyl)-2-(4-sulfophenyl)-based NPs showed effects on the PC3 cell lines, the efficiency of which was tested using 2H-thermorestetrazolium (MTS) and apoptotic assays. These NPs might also be effective in cancers treatment due to their easy access to delivery sites and their capacity to release the drug in response to variations in internal factors, such as, pH, effects of the surrounding chemicals, and other physiological indicators [70].

#### 2.3.4. Poly-(N,N-dimethyl)acrylamide: Conductive Co-Polymeric Material

N,N-dimethyl acrylamide, a useful hydrophilic monomer for drug delivery [71], was also formulated. Poly(N,N-dimethyl)acrylamide(PDMAAm)-coated maghemite (PDMAAm-coated-γ-Fe_2_O_3_) NPs were used for targeted drug delivery [72]. Colloidal and stable magnetic NPs containing DOX-linked co-polymer N,N-dimethyl acrylamide and/or N-acryloyl glycine methyl ester, or N-acryloylmethyl-6-aminohexanoate (Dh = 336 nm) were used. The carbodiimide was used to bind the co-polymers’ terminal carboxyl groups with the drug, alendronate, to treat glioblastomas, the most frequent and aggressive brain tumor. The methyl ester groups were subsequently transformed to hydrazides, where hydrolytically labile hydrazone bonds were used to bind the DOX. Bisphosphonate terminal groups were used to bind the polymers to the SPIONs, and finally, the anticancer impact of the DOX-conjugated nanoparticles were studied in the U-87 glioblastoma cell lines in terms of the particle’s internalizations and cells viability, which dropped to nearly zero at a concentration of 100 mg of the particles per ml. These findings demonstrated that poly (N,N-dimethyl) acrylamide-coated magnetic nanoparticles (MNPs) can effectively transport DOX to glioma cells [73]. In another investigation, a novel multifunctional, biocompatible, and water-soluble polymer ligand, dodecane-thiol-polymethacrylic acid (DDT-PMAA), was prepared as part of the co-precipitation method. The method was used to synthesize monodisperse, superiorly water-soluble MIONs (magnetic iron oxide nanoparticles), DDT-PMAA. The DDT-inherent PMAA’s features allowed it to manage the size of the MIONs, while simultaneously providing the MIONs with better and improved water solubility, long-term stability against aggregation and chemical oxidation. It also provided biocompatibility as well as a rich density of multifunctional surfaces having thioether and carboxylic acid group endings. The polymer ligand’s MW and the end groups’ concentrations were tuned to create ultra-small (~15 nm) MIONs with high magnetization (50 emu/g) value. The MIONs produced with 1.5 mM (milli moles) DDT-PMAA (5330 g mol^−1^) were extremely stable in solution and as dry powder over a long periods of time. At room temperature, the MIONs had a high degree of mono-dispersity and were superparamagnetic in nature. GPC, ^1^H-NMR, DLS, TEM, FT-IR, Raman, XRD, TGA, and VSM were used to analyze the polymer ligand and the MIONs–polymer nanomaterials. The cytotoxicity of the prepared MIONs was also tested using the MTT assays in order to demonstrate its bio-applications, and they were found to be non-toxic to normal cells and biocompatible. Finally, the MIONs were conjugated to the DOX, and the efficacy of the nano-system as a model drug delivery system was tested in HepG2 cells. The drug-MIONs conjugates, such as, covalently bound DOX-MIONs, and electrostatically loaded DOX-MIONs, were found to be significantly more efficient than the free-DOX alone [74]. Furthermore, a new thermo-responsive fluorescent polymer (TFP) was conjugated to the surface of the iron oxide MNPs for solid tumors. The TFP-MNPs were prepared through co-polymerization of the poly-(N-isopropyl) acrylamide (P-NIPAM), allyl amine and a biodegradable photo luminescent polymer. Afterwards, they were conjugated to the MNPs with the free radical polymerization technique. The in vitro cell-based experiments were used to assess the nano-preparations’ cytocompatibility, uptake, and cytotoxicity. Finally, the subcutaneous tumor xenograft mice models were used to conduct in vivo imaging, and treatment effectiveness of the preparation. The colloidal stability and superparamagnetic characteristics were maintained in TFP-MNPs, which were produced with a diameter of 135 nm, and zeta potential at −31 mV. These NPs were also taken up by the prostate cells, and skin cancer cells, in a dose-dependent manner at 41 °C. The tumor cells were killed effectively. The obtained findings suggested that the TFP-MNPs have the potential to be used as multifunctional theranostic NPs in a variety of biological applications, including the treatment of solid tumors [75]. Therefore, SPIONs, having ideal characteristics, were utilized as part of the magnetically driven, targeted drug delivery carrier and contrast probe. Novel MagAlg(condensed colloidal nano-crystal clusters)-DOX systems were prepared, and tested under in vitro conditions on mouse fibroblasts and breast cancer cell lines. They showed significant outcomes. The cytotoxic effects of the MagAlg-DOX combination showed a late onset and the effects were delayed in both the cell lines, as compared to the free-DOX formulation. These observations were attributed to the drug’s strong binding to the nanomaterials, as well as to the diverse ways the DOX and MagAlg-DOX were internalized into the tumor cells. The NPs were shown to reduce and eradicate the effects of DOX, particularly more so in the MCF-7 cancer cell lines. Thus, the cytotoxic effects of DOX-loaded SPIONs in comparison to free-DOX on healthy and cancer cell lines were observed, together with the observations of the gene expression levels that resulted from the formulation change, and indicated a different biomechanistic with the NP clusters [76].

### 2.4. Thermo-Responsive Polymer Conjugates

The field of thermo-responsive polymers has been rapidly growing, and considerable work has gone into developing temperature-sensitive macromolecules that can be used to create new smart materials [70]. The HmSiO_2_ (hollow mesoporous silica)-based thermo-responsive polymer embedded with Fe_3_O_4_-type SPIONs with DOX were prepared. In the presence of SPIONs, oxidant and cross linker, in situ polymerization of the NIPAM (N-isopropyl acrylamide) and MAm (methacrylamide) monomers produced hollow mesoporous silica based NPs. The mesoporous silica (HmSiO_2_) was covered with P(NIPAM-MAm) polymer, and Fe_3_O_4_ NPs, denoted as (F), to produce the functionalized, (F)-embedded nano-composite, HmSiO_2_-F-P-(NIPAM-MAm) nano-platform. The encapsulation efficacy of the HmSiO_2_-F-P(NIPAM-MAm)-DOX was determined at 95%. The release profile was found to be pH responsive and temperature dependent. In vitro cellular uptake investigations proved the ability of the HmSiO_2_-F-P(NIPAM-MAm)-DOX nanocomposite-based nano-carrier to integrate into the HeLa cells, and the in vitro cytotoxicity experiments against HeLa cells established that the nano-composite had high anticancer activity [77]. In another study, thermo-responsive core/shell MNPs with an iron oxide core and a thermo-responsive co-polymer shell comprising of 2-(2-methoxy)-ethyl methacrylate (MEO_2_MA) and oligo(ethylene glycol) methacrylate (OEGMA) moieties were investigated. These smart nano-entities with the magnetic capabilities of the core were merged with a drug carrier in which the chemotherapeutic agent, DOX, was loaded into thermo-responsive MNPs through supramolecular interactions. The core/shell MNPs were tested for their cytotoxicity against the SKOV-3 cell lines for human ovarian cancer, and the polymer-capped MNPs showed practically no toxicity at concentrations up to 12 g mL^−1^. However, when loaded with DOX, there was an increase in the cytotoxicity and a decrease in the SKOV-3 cells’ viability. The study concluded that these SPIONs with stealth features can transport and deliver non-cancer chemotherapeutic drugs to tumors and have the potential to be used in multimodal cancer therapy [78].

### 2.5. PAMAM-Dendrimer’s Conjugate

Dendrimers have often been used as a drug delivery option since the late 1990s, and their efficacy has been confirmed in enhancing the solubility, stability, and oral bioavailability of a variety of drugs and other deliverables [79]. A pH-responsive drug release system was developed using mPEG-G2.5 PAMAM dendrimer, and the DOX was conjugated with the SPIONs for the transport of DOX to the tumor through enhanced permeability and retention (EPR) effects established under lysosomal pH (pH = 5.0) conditions. The DOX-conjugated SPIONs showed the potential to enhance the effects of MRI contrast agent, and provided better cancer management when the chemotherapeutic agent, DOX, was delivered to the target site. Despite the lack of targeting ligands on the surface of the dendrimer–DOX-loaded SPIONs, the nano-structures were found to be beneficial for in vivo cancer detection and delivery of the drug [80].

### 2.6. Zwitter Ionic Polymer Conjugates

The zwitter ionic polymers holding both positive and negative charges in their structures have great potential in nanotechnology, biomaterials science, and nanomedicine [81]. The polymers have been suggested to resist non-specific protein adsorption, bacterial adhesion, and biofilm formation [82] and are considered biocompatible. They can also be part of in vivo drug delivery systems, and used as non-fouling coatings of biomedical implants, blood contacted sensor making, as part of a separation membrane, and in various coatings, including for the surface coating of NPs. The zwitter ionic polymers have been suggested to suppress protein adsorption at the surface of NPs in complex biological media [83], and have been demonstrated to inhibit formation of protein corona in in model albumin solutions and in whole serum media. The non-formation of irreversible and hard, as well as reversible and soft, protein corona around the model’s NP systems with a coating of sulfo-betaine, phosphoryl-choline, and carboxy-betaine polymer ligands have been demonstrated. The NPs’ sensing and tracking in live cells cytoplasm corroborated the in vitro findings. Further developments have led to efficient intracellular delivery of the attached/encapsulated payload materials by the zwitterionic polymers. 

Nano-structured delivery systems, which allowed for the efficient delivery of the DOX drug payload for achieving therapeutic targets, are an important advancement in specialized and site-specific drug delivery options. By carefully modifying the structural properties of the NPs, the drugs placed on/into the SPIONs have been successfully site directed and selectively delivered to various cancer locations. Table 1 summarizes the synthetic polymeric entities conjugated to SPIONs for DOX delivery together with their structural and functional details. 

### 2.7. Natural Polymer-Based DOX-SPIONs Conjugates

#### 2.7.1. Dextran

Dextran has long been used as a temporary plasma replacement entity due to its high biocompatibility. The dextran polysaccharides possess multiple star-like arms with a cyclodextrin core. The aliphatic chains added to the star-like dextran structures allowed the amphiphilic polymers to self-assemble into nanoscale micelles in water at concentrations significantly lower than their linear structural analogs, resulting in stable NPs in the aquatic media. The SPIONs clusters wrapped in amphiphilic dextran polymer have been used as an effective magnetic resonance imaging probe. On a 1.5 T clinical MRI scanner, the resulting SPIONs formulation exhibited a high T(2) relaxivity of 436.8 Fe mM^−1^S^−1^), and the dual functional probes were created using polymeric micelles containing both the SPIONs and DOX, which were utilized for treatment and probing purposes [84].

#### 2.7.2. Chitosan

The diverse physicochemical features, including biodegradability, biocompatibility, and non-toxicity have catapulted chitosan-based nanomaterials to the forefront of nano-theranostic applications, and the polymer has attracted much interest owing to their versatility in biological applications practiced for a long time [85]. The DOX-loaded and chitosan-coated SPIONs (Fe_3_O_4_ types) with outstanding encapsulation efficiencies were produced using the co-precipitation and emulsification cross-linking methods of NPs preparations. The chitosan-loaded SPIONs (0.5 mg/mg; *p* ≤ 0.05) had a greater drug-loading efficacy for the DOX (3.2 mg/mg NPs) than the naked ones. In A2780 and OVCAR-3 human ovarian cancer cell lines, with significant IC_50_ (2.0±0.6 and 7.1±2.7 mm DOX), and IC_90_ (4.0±9.2 and 10±0.5 mm DOX) values were found after 96 h of incubation with DOX-loaded, and DOX-loaded-chitosan-coated-SPIONs under the initial trial study. The A2780 and OVCAR-3 cell lines demonstrated 95% and 98% growth inhibitions, respectively, after 96 h of exposure to DOX-chitosan-SPIONs (*p* ≤ 0.05). After a 5 day (120 h) incubation with DOX-chitosan-SPIONs, the A2780 and OVCAR-3 cell lines absorbed 120 and 110 pg iron/cell, respectively, (*p* ≤ 0.05), thereby establishing the superior working efficiency of the nano-preparation [86]. 

#### 2.7.3. Chondroitin-4-Sulfate

Chondroitin sulfate (CS) is a naturally generated bioactive macromolecule that has gained much interest owing to its bioactivities. It is also used extensively for the purpose of drug delivery [87]. SPIONs were developed using glycosaminoglycan chondroitin-4-sulfate, and were used to load DOX, which was included into the formulation up to 2% (*w/w*) due to the fact of its physical interaction with the CS, and the vibrational magnetometry revealed the superparamagnetic nature of the resultant nano-preparation. In an in vitro release profile at pH 7.4, 96.67% of the DOX was released after 24 h (first-order kinetics) of injunction. The DOX cytotoxicity in the SPIONs formulation was significantly higher (*p* ≤ 0.0001) than the free-DOX solution’s cytotoxicity, as observed in the MCF7 cancer cell lines (IC_50_ values 6.294 ± 0.4169 and 11.316 ± 0.1102 µg.mL^−1^, respectively) [88]. 

#### 2.7.4. Hyaluronan

Hyaluronan (HU), a natural polysaccharide, has been used as a potential ligand for cancer-targeted NP-based drug deliveries [89]. The expression of IL-6 in triple-negative breast cancer is higher than in healthy breast tissue, and there is a significant relationship between the inflammatory response, tumor growth, and cancer metastasis. A FACS (fluorescence-activated cell sorting) analysis was used to quantify the effects of DOX-HU-SPIONs formulated for the purpose of apoptosis in MDA-MB-231 cell lines. The cells treated with DOX alone, or DOX-HU-SPIONs were compared, and an ELISA (enzyme-linked immunosorbent assay) test was conducted to determine the nitrate levels in the conditioned media. The pro-inflammatory and anti-inflammatory cytokine (IL-6 and IL-10) levels were also investigated. A confocal imaging experiment revealed that the DOX-HU-SPIONs exhibited greater cytoplasmic uptake than the free-DOX. The DOX-HU-SPIONs boosted apoptosis and significantly reduced both pro-inflammatory mediators, IL-6 and NF-B, as compared with the free-DOX alone. The anti-inflammatory mediator, IL-10, and nitrate secretion levels in the DOX and DOX-HU-SPIONs were not lowered. According to the findings, DOX-HU-SPIONs-based drug delivery indicates strong potential for the treatment of metastatic and chemoresistant breast cancers by increasing the therapeutic efficacy of the non-cancer drug, and also lowering the off-target effects of the drug [90]. 

#### 2.7.5. Starch-Octanoic Acid Micelles

MRI-based diagnosis and high-efficiency tumor targeting are crucial components in cancer chemotherapy. Starch-octanoic acid (ST-OA) micelles (m) (ST-OAm) were prepared using the hydrophobic core of the core–shell micellar structures to co-encapsulate the DOX, and the SPIONs [91]. The SPIONs were coupled with a biopolymer, polyaspartamide (PA), to create a biological construct to be utilized for cancer detection, monitoring, targeting of the drug, and eventual therapy. The PA was coupled with the biotin and DOX through their functional groups to produce a multifunctional polymer-based material for enhancement of the targeting of cancer cells and their annihilation. The superparamagnetic properties of the SPIONs with evenly distributed average spherical diameters of roughly 10 nm were found to be effective in enhancing the transverse 1/T 2 relaxation rate and darkening of the T 2-weighted MR picture for cancer detection by MRI. An in vivo study on tumors revealed that injecting the bio-construct three times more than the un-injected controls slowed the growth of tumors, as compared to the un-injected controls [92].

#### 2.7.6. Heparin

The chemical and biological characteristics of the heparin (HP), a biological polymer, as related to the NPs’ preparation and use, have been explored in a variety of biological processes [93]. DOX-conjugated heparin was coated on SPIONs, producing the preparation, DH-SPIONs, with an average NP size of 102 nm. The preparation was used as an MRI contrast agent, and for the drug’s targeted delivery to cancer cells. The zeta potential of the DH-SPIONs was very high. Prussian blue staining, total iron contents, in vitro MRI imaging, and TEM analyses were used to demonstrate the superparamagnetic clustering effects of the DH-SPIONs nano-formulation. The DH-SPIONs were also shown to be highly effective, more so than the free-DOX alone, at reducing the tumor size and extending the lives of the mice. These findings pointed to the usefulness of the DH-SPIONs as a potential nano-preparation for cancer therapy, and its possible clinical diagnosis [94].

#### 2.7.7. Albumin

Albumin has long been thought of as a viable material for NP production and is considered suitable for application in bioimaging and drug delivery, owing to its animal origins. It is biocompatible, biodegradable, and is devoid of untoward effects [93]. The use of nano- and micro-scale albumin carriers with encapsulated Fe_3_O_4_ and DOX, which could be concentrated upon delivery at a predefined site in the in vivo situations through control transport by a magnetic field, was one of the first trials of the magnetic targeting in chemotherapy. The local accumulation of DOX achieved through this type of delivery mechanism was as comparable to as that achieved with a 100-folds larger dose of the free-DOX alone [95]. The bovine serum albumin (BSA) mediated drug delivery was explored using multi-spectroscopic methods, and molecular modeling calculations were performed to obtain a full evaluations of the biosafety of the DOX-SPIONs entangled albumin nan0-preparation. The DOX-SPIONs-albumin-based delivery unraveled the framework conformations of the BSA and led to alterations in the microenvironment of the amide moieties, as seen through the ultraviolet absorption and synchronous fluorescence findings. The contents of the helix were reduced from 68.62% to 62.76%, as measured using the circular dichroism (CD) chiroptical technique, which indicated the presence of quantifiable alterations in the protein’s secondary structures. The quenching mode was determined to be the static contact, thereby generating a stable bioconjugate using Stern–Volmer analysis. The DOX favored highly polar binding sites in the exterior portions of the BSA protein’s domain, as also shown in the molecular modeling, where the presence of hydrogen bonds were of high importance. The study also showed that BSA-mediated DOX-SPIONs drug delivery system has a negative impact on the protein frame’s conformation, which also altered its physiological functions [96]. 

#### 2.7.8. Spider Silk

Spider silk, that has been bioengineered, offered excellent mechanical properties, biocompatibility, and biodegradability. The EMS2 (spider protein spheres) had the highest concentrations of the SPIONs. The SEM, EDX, SQUID (superconducting quantum interference device), MIP-OES (microwave-induced plasma with optical emission spectrometry), and zeta potential measurements confirmed the presence of magnetite SPIONs in these carriers. The interaction of EMS2 and SPIONs had no effects on the superparamagnetic properties of the SPIONs, but it did influence the secondary structure of the silk-based spheres. For both the EMS2 and EMS2-SPIONs, the drug showed a pH-dependent release profile with better kinetics for the EMS2-SPIONs drug carriers. The spheres have been utilized in experimental cancer bioactivity tests that combined heat-based drug delivery option [97].

#### 2.7.9. Prostate-Specific Membrane Antigen Aptamers

The prostate-specific membrane antigen (PSMA) aptamers, conjugated thermally and cross-linked (TCL) with SPIONs (TCL-SPIONs), were pressed in relation to diagnose the prostate cancer under in vivo conditions. The materials were also used to deliver the DOX to malignant cells through selectively using the MRI technique. The PSMA functionalized through hybridization and produced through TCL procedure attached the SPIONs to produce Apt-hybr-TCL-SPIONs, which showed preferential binding to the targeted prostate cancer cells (LNCaP) under in vitro and in vivo conditions with T(2)-weighted MRI imaging. The DOX-Apt-hybr-TCL-SPIONs showed selective drug delivery efficacy in an LNCaP xenograft mouse model. On preparative levels, after the DOX molecules were loaded onto the Apt-hybr-TCL-SPIONs through intercalation of the DOX to the CG-rich duplex, containing PSMA aptamer, the electrostatic interactions between the DOX–polymer coated layers of the NPs facilitated the DOX delivery. These results suggested that the DOX-Apt-hybr-TCL-SPIONs have the potential to be employed as a new cancer nano-theranostics for prostate cancer [98].

### 2.8. Capped SPIONs-Based DOX Conjugates

#### 2.8.1. Citric-Acid-Capped SPIONs-DOX Conjugate

Among the use of small molecules as capping materials, citric acid has been utilized as part of the NP platforms for facilitated DOX delivery [99]. Water dispersed samples of citric-acid-capped SPIONs were employed to bind the DOX to produce acid-capped SPIONs-based NPs through non-covalent physicochemical interactions. The cellular uptake of these NPs was shown to be at considerably higher levels, but with magnetic targeting and monitoring, it was made to perform better. The drug-free NPs were shown to be safe for the cells; nevertheless, the drug conjugation caused drug-induced toxicity due to the NPs’ prolonged residence and extended release of the cytotoxic drug, DOX from the nano-preparation [100]. 

#### 2.8.2. Folic-Acid-Capped SPIONs-DOX Conjugate

Another acid molecule, folic acid (FA), targeted to the surface of cancer cells by the overexpressed-folic acid receptor (FR), was conjugated to NPs in a straightforward manner to improve the FR-mediated, and targeted delivery of chemotherapeutic agents, including DOX [101], to the receptor site. The manufacture of folic-acid-linked galactoxyl-O-glucan-SPIONs (FA-SPIONs) for the loading and controlled release of encapsulated DOX removed the bottlenecks associated with SPIONs-based DOX delivery and improved drug delivery and its efficiency. The DOX-FA-SPIONs, as constructed, caused a dose-dependent increase in cytotoxicity. The phenomenon was observed in folate receptor-positive (FR+) cells through a Caspase-mediated programmed cell death, as demonstrated in reverse with the free-DOX and in the non-targeted delivery. The nano-preparation also showed no toxicity to the normal cells/organs [102].

### 2.9. SPIONs Monomer Conjugate: Trimethoxy Silylpropyl Ethylenediamine Triacetic Acid

As a DOX carrier for glioblastoma multiforme (GBM) therapy, biocompatible SPIONs were prepared and stabilized with trimethoxysilylpropyl ethylene diamine triacetic acid (EDT). The DOX-EDT-SPIONs were prepared by loading the DOX onto the EDT-SPIONs, and from the nano-preparation, the drug was considerably fast released in an acidic microenvironment within four days of nano-scale drug administration, as compared to the free-DOX alone. The DOX-loaded-EDT-SPIONs (DOX-EDT-SPIONs) exhibited excellent absorption in the mouse’s kidneys, and were transfected with multidrug resistant protein-1 (MDCK-MDR1) and human U251 GBM cells. The DOX-EDT-SPIONs also increased the DOX absorption in U251 cells by 2.8-folds, and considerably decreased the growth of U251 cells. The DOX-EDT-SPIONs also improved the DOX’s permeability across the MDCK-MDR1 monolayers, as compared to the free-DOX alone. The cytotoxicity in U251 cells was similar in both the treatment groups. Using a cadherin-binding peptide (ADTC5) to temporarily open the tight junctions in combination with an externally applied magnetic field, a dramatic improvement in DOX-EDT-SPIONs’ permeability and cytotoxicity was observed, including in the MDCK-MDR1-GBM co-culture model [103].

### 2.10. SPIONs-Miscellaneous Entities DOX Conjugates

#### 2.10.1. Graphene Oxide

Graphene oxide (GO) has sparked the interest of biomedical community as a promising platform for biological sensing, in vitro fluorescence imaging, and tissue-structure sensing. Recently, SPIONs-based graphene oxide entity was developed, and used for DOX delivery to the MCF-7 breast cancer cell lines. It was observed that the DOX-loaded nano-carrier improved the cytotoxic effects of the DOX on the breast cancer cell lines, as compared to the free-DOX alone. These results introduced the possibility of a DOX-loaded carrier as a potential platform for in vitro targeting of breast and other cancer cells [104]. The GO was combined with the SPIONs, which were found to be biocompatible for magnetically driven drug delivery system, and they were also useful as a magnetic resonance contrast agent for MRI. With an average size of 260 nm, in the larger size domains, the cytotoxicity of the GO-Fe_3_O_4_ conjugates were comparable to that of the GO, and the r2/r1 relaxivity ratio of the Fe_3_O_4_ NPs was found to be 10.7. As part of the construction of the nano-entity, the preparation provided magnetically targeted drug delivery options through the intermediacy of the SPIONs. The GO-Fe_3_O_4_ demonstrated significant loading and a 2.5-folds increase in the drug’s effectiveness at transporting it, which was non-covalently coupled to the GO. The phenomenon was tracked through fluorescent imaging [105]. A simple and non-covalent synthesis of GO-based functional hybrid material prepared with gold NPs, and fluorescently labeled SPIONs (GO-MNcy5.5-AuNP) was also prepared to improve the magnetic relaxivity of the SPIONs-based nano-systems. The cy5.5 fluorescence was completely quenched, and the gold NPs’ surface plasmon peak at 520 nm was identified in the GO-MNcy5.5-AuNP nano-hybrid, which had all of the physical attributes of each constituent component. The hybrid nanomaterial had an ultrahigh DOX loading capacity of 6.05 mg ml-1 of drug concentration. The prepared nano-hybrid material showed potential as a promising platform for theranostic agents owing to its contrast capabilities and anticancer drug loading efficiency [106].

#### 2.10.2. Hydroxyapatite

Hydroxyapatite (HA) possesses a variety of physicochemical features that lists it as a promising choice in nano-formulations for drug delivery purposes. Its biocompatibility and biodegradability, together with its relatively simple synthetic protocols for the fabrication of NPs of specific sizes and shapes, as well as the prepared materials prompt response to environmental stimuli, have made these structures a prominent material in nano-scale drug delivery and diagnostic applications [107]. pH-responsive multifunctional delivery carriers for drugs were constructed using mesostructured HA coatings with a SPIONs base. Large pores in the produced mesoporous material served as an effective DOX carrier with a loading efficiency of ~93%, a much higher ratio than that of the regular HA-based NPs. The drug release increased dramatically from ~ 10% to nearly 70% of the adsorbed drug, when the pH of the release medium (PBS based) was altered from pH 7.4 to pH 5.5. The free-DOX incubation reduced the viability of the SKBR3 and T47D cell lines by 54.7% and 57.3%, respectively. These observations were found to be very close to the 56.8% and 60.4% deliveries, as observed with the free-DOX alone [108].

#### 2.10.3. Iodinated Oils

This new drug-delivery method, which uses SPIONs and iodized oil (IZO), was found to be viable, and successful at increasing selective intra-arterial (IA) delivery to an experimentally produced hepatic malignancy in rabbits. The group A (DOX alone, *n* = 3), group B (DOX-IZO, *n* = 3), group C (DOX-SPIONs, *n* = 4), and group D (DOX-SPIONs-IZO, *n* = 5) were tested. At the tumor locations, the animals in groups C and D had considerably lower magnetic resonance signal intensities than the animals in groups A and B, which inversely correlated to the SPIONs’ deposition. The animals in group D had the lowest serum levels of DOX and low DOX transit into the systemic circulation, which was at increased levels at and for 180 min after the medication’s delivery. The animals in groups A, B, C, and D had intratumoral DOX concentrations of 72.4, 142.0, 264.1, and 679.6 ng/g, respectively. In the animals in group A, 65.3% animals were alive, while 1.3% animals were alive in group B, 17% in group C, and 0.1% animals were alive in the group D. Thus, the drug delivery system designed through the use of SPIONs and IZO for drug delivery to the liver cancer resulted in better drug targeting. These findings called for a detailed study to establish the procedure as part of a proper therapeutic module [109].

Table 2 summarizes some of the natural polymers, graphene oxide, and other monomeric molecules, as well as miscellaneous products used in the SPIONs’ conjugation to facilitate the anticancer drug DOX’s delivery to cancer sites under in vivo and in in vitro conditions.

## 3. Polymer Comparatives: Nano-systems Sizes, Drug Loading, Zeta Potential, and Stability

Among the prime concerns regarding SPIONs-polymeric and non-polymeric conjugates with an incorporated drug, are the shelf-life and the transitional stability of the prepared medication during the drug delivery process. The nanomaterial’s size plays an important role in eliciting the stability of the prepared conjugates and the metal core. The metallic core magnetization’s increase, or in other terms the saturation in magnetic properties, is known to lower the stability of the particles [111]. The wrapping around the metal, necessary for safe and effective drug delivery, also lowers the magnetic effects of the prepared nano-conjugates. The zeta potential manifests the stability factor and is considered viable in both the positive and the negative modes of measurement within the median values of 40–60 mV. Smaller sized, spherical particles and nano-preparations are considered to be stable, and the zeta potential, a dispersion stability indicator, together with the other parameters of pH, ionic strength, concentration of the medium [112,113,114,115], and polydispersity contribute to the stability of nano-systems composed of metallic NPs and their conjugates. Lower sizes of the nano-preparations have been favored; nonetheless, a median size under or around 100–150 nm has been considered better for loading larger drug quantities for effective and dose-maneuvered delivery. A comparison of the different parameters, including size, zeta potential, drug loading, and drug release of the different polymer-based conjugates is presented in Table 3. A direct comparison of the polymer-based conjugates’ sizes revealed the better characteristics of the PLA-PEG conjugate for DOX delivery. The nano-conjugates were moderately sized at ~45 nm, with a zeta potential of −25.6 mV and ~80% drug loading, and 92% drug release in 2 h, although this was a fast release. Among the other conjugates, the PEGylated silica based, PLGA-PEG 4000, and chondritin-4-sulfate based nano-carriers are worth mentioning. 

## 4. Polymer Conjugates Comparatives: Cell Lines and Cytotoxicity

Table 4 summarizes the nano-conjugates’ cell viability assays against a number of different cell lines. The lowest cell viability was observed for the PEG-PAMAM, graphene oxide (GO), GO-APTES conjugates against Caco-2 and HeLa cell lines. Among the nano-conjugates active against the breast cancer cell lines MCF-7, the GO-β-CD conjugate with 50% cell viability, and a 1 μM, IC_50_ value were observed. 

In a nutshell, various techniques for the manufacturing and functionalization of SPIONs have been developed, together with their functionalization with inorganic and organic molecules. The *f*-NPs have been explored for the stabilization of magnetic colloidal suspensions, which is especially significant, if any medicinal use is planned. The SPIONs and other types of MNPs have also been used as MRI contrast agents, as part of effective drug delivery systems, and for imaging/sensing purposes. However, the limited solubility and biodegradability of SPIONs and other iron oxide and MNPs need improvement for magnetization efficiency with safer applications, and workability for single/combined targeted transport of drugs to desired cancer sites through magnetic hyperthermia, which importantly forms the part of the non-cancer agents’ non-invasive and safe chemotherapeutic applications. Body-surface located cancers (e.g., skin squamosal cells carcinoma) and relapsing cancers (e.g., benign and crucial malignant melanoma, Kaposi’s sarcoma, and metastatic cancers including cancers of breast, liver, cervix, prostate, ovary, and lungs) have been targeted by SPIONs-based treatment modules in efficient manners. Nonetheless, due to the functionalized/coated/encapsulated SPIONs, a large payload carrying capacity, multi-functional behavior, and multi-modal imaging capabilities, thus generated, have provided these nano-systems an important place in theranostic platforms for administration of image-guided cancer treatment development as part of cancer management therapy at broader scales of cancer types handling. Nano-preparations of SPIONs with both covalent and noncovalent connections between the polymer matrix and the SPIONs have been used in a variety of biomedical applications, including contrast detectors, theranostics, colloidal nano-crystal clusters, and star-like polymers for imaging, gene and drug deliveries, and in tissue engineering [117]. The unique combination of superparamagnetism and pH-responsive characteristics have further provided promising foundations for building an innovative biocompatible drug carrier for the targeted delivery of drugs in cancer treatments. By combining magnetically accelerated convective diffusion, the DOX-SPIONs have certainly increased the efficiency in GBM treatment, which for larger part have been elusive. 

## 5. SPIONs-Inspired Magnetic Spectroscopy: Development of a Medical Diagnostic, Tomographic Imaging, and Quantification Tools

SPIONs have been part of the imaging modality nearly since it discovery and inception in oncological applications. The dual advantage of treatment through killing cancer cells and characteristic of magnetically driving the loaded (conjugated/encapsulated) payload of drug towards the cancer site has opened the ways for further advancements. Notably, the SPIONs and gadolinium NPs (Gd-NPs) have been at this forefront. The potential to complement the therapy and diagnosis, as a theranostic agent, together with other imaging modalities have enhanced the prospects of SPIONs as being part of clinical imaging module, and this inherently has the potential to obtain high definition 2D and 3D constructed visions of the image, supported by the appropriate computing and software, in comparison to a single-sided configuration presenting a 1D image, which is inherent to spatially inhomogeneous imaging. Spatial field free point (FFP) versus spatial field free line (FFL) imaging has also been achieved. Moreover, the hardware to scan have also been under constant upgrade in imaging modality’s functional characteristics and image enhancement tools, together with the computographics and the requisite software for signal enhancements, noise suppression, and better vision developments [118] of the region of interest in imaging. Magnetic particle imaging (MPI) for angiography and chronic kidney malfunctions [119], use of amine-functionalized MNPs [120], and nebulized MNPs use in pulmonary tracing [121], in essence, are dependent upon the nano-scale tracer materials’ properties optimized to detail the affected organs’ disparity against normal cells. Iron oxide nano-scale tracers with a multi-folds signal-to-noise ratio with enhanced spatial resolution have also been developed. The metallic nano-tracer also needs to be biocompatible, and preferably biodegradable, as well as need to have a reasonable residence time to record the imaging [122]. In this connection, the use of magnetic particle spectroscopy (MPS) to determine the targeting efficiency has been reported [123]. MPS, also known as magnetization response spectroscopy (MRS), was primarily derived from the MPI phenomenon due to the MNPs’ static and dynamic magnetic properties. Because of the non-linearity of the magnetization curves, a detection signal can be recorded from a single and gradiometric induction coil. In MPS, a sinusoidal signal with high frequency and large amplitude is applied to the MNPs sample. The resultant spectrum is obtained in response to excitation frequency versus the receiving frequency. The method can be regarded as a zero-dimensional magnetic particle scanner wherein a drive field and a receiver coils are provided, but with no possible spatial encoding, unlike a multidimensional scanner, which is a desirable requisite. Herein, the particle magnetization derivative is measured by the receiving coil. The MPS, which was fundamentally designed for SPIONs and other applications, including magnetic fingerprinting for target tracking, and the target’s magnetic property-based identification, has evolved as a sensitive procedure for biomedical assays, cell labeling, MNPs tracking, as well as blood analysis under magnetic influence probably owing to the heme based iron contents of the RBC (Red Blood Cells). The hyperthermia, and magnetic imaging have also been possible, a later development, due to the high spatial and temporal mappings with the help of temperature and viscosity gradients. Thus, the current instruments recording the MPS, functionally operate at 25 kHz/40 mT maximal oscillating excitation, and a maximal 40 mT offset magnetic field strength frequency value, although a higher excitation frequency (100 kHz) with higher amplitudes and static background fields’ instruments are under development. The current MPS spectra are analyzed as an approximation using Langevin function model extended by Debye pre-factor accounting of MNPs’ magnetic properties. Because MNPs experience varying magnetic field strengths at different locations, the strength reaching the receiver coil is dependent on its sensitivity, which leads to spatial-dependent amplitude, wherein the sensitivity and field strength need to have errors < 1%, since the physical models for the MPS are very sensitive towards the drive field, and the amplitude disparity. In this situation of higher sensitivity, the error margins are of crucial consequence, and electrical and magnetic designs are tough to achieve for a super-compatible machines, at least in very near future. Nonetheless, the technique has opened the SPIONs-based drug delivery and imaging for better analyses, and improved oncological results. A technical confluence of MRI and MPI, under magnetic fields gradients, have been adopted for magnetic particles quantification (MPQ) of MNPs samples. A responsive concentration measured out from serial dilutions of MNPs in two different media, together with stronger effects of surrounding media on the MRI responses, as compared to the MPI performance were analyzed. The strong MRI responses validated by the MPS, showed comparable imaging commonalities, and responsive concentration range signals, which helped towards standardization and quantification of the MNPs. The magnetic drug targeting (MDT), through an in vitro flow phantom of the MPS instrument provided reproducible and defined MDT signals, wherein the tubular part of the MPS flow phantom, directed through inside of the detection/receiver coil of the MPS instrument determined the targeting efficiency. The MPS offered a temporal resolution of seconds for the MNPs sample, and an outstanding specific sensitivity at nano-gram scale of iron. The magnet specifications, geometries, and the tube specifications were employed in reference to various physical parameters (i.e., diameter, flow rate, strength and magnetic targeting gradient) to generate the MNP data needed for quantification. For both MRI and MPI, Resovist^®^ has been used as SPIONs-based contrast agent. A current and comprehensive review on MNPs applications [124] and scanner developments [125] is available for more details. 

## 6. SPIONs-DOX Conjugate: Nano-theranostic Agent in Diagnosis and Therapy

Theranostic nanoparticles (TNPs) have the potential to open up new vistas in the field of individualized drug delivery managements as part of the personalized medicine. The major advantages rests in their dual functionality as the imaging and treatment module. Chitosan NPs were employed as a dual-action nano-carrier for the DOX delivery using ionic gelation process (SPIONs as the imaging agent). The DOX-SPIONs loaded into an ACSD (affinity capture surface display assay-based) matrix in differing quantities revealed drug’s burst release at pH 5.5, with continuous release of the drug at pH 7.4, thereby also suggested a pH-sensitive drug release characteristic. When iron concentration as part of the SPIONs was raised for the in vitro experiments, the T2 relaxation periods were found to be shorter as detected by magnetic resonance imaging (MRI). The absorption of NPs at the optimum dose was validated by MRI imaging of C6 glioma cells, and the ACSD-NPs was easily used as a theranostic formulation for the diagnosis and treatment of glioblastoma [110]. In the realm of nano-theranostics, SPIONs (polysaccharide-decorated-SPIONs) such as carboxy-methyl-based Assam-bora rice starch (ABRS) stabilized SPIONs (CM-ABRS SPIONs) have shown potential for magnetic drug targeting using the co-precipitation method. According to spectrofluorometric investigation, the electrostatic interactions allowed the DOX to be loaded up to 6% (*w/w*) onto the CM-ABRS SPIONs. A docking study against overexpressed receptors (HER-2, and Folate receptors) in cancer cells, investigated the molecular level drug performance of the DOX-CM-ABRS-SPIONs, which revealed promising results as compared to the standard, free-DOX solution [116]. The therapeutic efficacy of the non-covalently linked hydrogel network created by using cucurbit-[7]-uril as a supramolecular linker to attach the superparamagnetic-Fe_2_O_3_ NPs (SPIONs) to the polymer backbone of the catechol-functionalized chitosan, exhibited vibrational mobility, and heat production, thereby endowing the hybrid supramolecular hydrogel both with thermos- and chemotherapeutic features [126]. A vessel mimicking the phantom model with live C6 glioma cells was used to demonstrate an increase of 5.4-folds in the drug’s targeting efficacy monitored by the magnetism-assisted targeting of the SPIONs-embedded droplets. It also showed an effective cell disruption, using the insonation-induced acoustic droplet vaporization (ADV) method, which indicates its potential for further development for future therapeutic and imaging needs [127]. Other examples of DOX, biotin, and polyaspartamide are also reported [111].

## 7. SPIONs and Polymeric Conjugates: Toxicological Issues and Biosafety Facts

### 7.1. Toxicological Issues

Safety issues concerning nanomaterials have gained center stage. The toxicity generated from metal and metal oxide NPs and polymeric nano-entities is well recorded [128,129]. Studies pertaining to various nano-forms toxicity and their adverse effects are now explicit. Both in vivo and in vitro toxicity evaluation methods, both in different animal models and cell-lines, have been employed.

The cytotoxic nanomaterials designed for the purpose of non-cancer agent, have the inherent capability to affect cellular processes leading to subcellular alterations including effects on cellular signaling, altered proteins production levels, changed gene(s) expressions, mitochondrial damages, enzymatic imbalances, haphazard cellular feed-back mechanism affecting the cellular function, histological non-compatibility, tissue and organ damages, increased cells viability, and eventual cell death. The effects of DNA synthesis and damage, immunogenicity, cell proliferation effects; changes in exocytosis, hemolysis, apoptosis, necrosis, and metabolic and oxidative states; dose-linked overt issues are some of the in vivo conditions based parameters for evaluations of the overall toxicity [130].

Toxicity manifestations have upper limits so as not to harm normal cells while also being effective against cancer cells and cancer cell lines. The characteristics of the nano-system colloids’ size, zeta potential, stability (shelf and organ transport), polydispersity, surface area, volume, cellular permeability, ability to evade the immune system, stability in the circulatory apparatus, intact arrival at affected cancerous organs, and interactions at the site and in the biosystem during delivery transitions as well as organs’ accumulation have posed enormous challenges concerning non-cancer nanomaterials, including nanoconjugates, towards their development as an effective delivery module and the effectiveness of a drug. The delivered drug’s outreached concentration at the site differs on the nano-conjugates’ affinity and accumulation at the site as well as any deposition of it, or the residual drug’s availability, all together tend to generate protein corona through the encountered biomolecules, especially circulating and at the site of available proteins’ adhesion to the surface of the exposed core nano-entity, especially the metal cores’ exposure.

For polymeric nanomaterials, especially the encapsulates and conjugates, the constituent polymers’ toxicity is dependent upon the chemo-biological properties of the polymer and its biocompatibility, biodegradation half-life; nano-modules’ size, shape, polydispersity, colloidal stability, tunable properties, and surface coating; payload delivery mode and method [131,132].

### 7.2. Biosafety Facts

In this context, the hazardous roles of metal NPs owing to their catalytic character, high reactivity, and other physicochemical properties, including magnetism and the hysteresis, together with extent of their exposure determines the risk [133]. The natural protective mechanisms of biosystems do not provide effective safety against nano-entities. The macrophages intake the polymer encapsulates and polymer conjugates, especially the PEGylated nano-entities more efficiently in comparison to the smaller-sized nanomaterials. Accumulation of nanomaterials is primarily considered responsible for much of the toxicity, and size of the nano-entity has substantial effects on their interactions with the cells. Nonetheless, the nanomaterials cellular absorption levels, surface features of the nanomaterials and the cells, cell type, and the intracellular localization (i.e., endocytosis and passive intake of nanomaterials irrespective of their size) leads to adverse effects and cytotoxicity [134]. 

Metallic NPs have facilitated intracellular absorptions, and pass through the tight biological, and other cellular junctions. The encapsulated and conjugated metallic NPs have been found to be quickly released from their ligands and activated as the naked nano-entity. Smaller NPs have shown longer t_1/2_ than larger ones [135]. Their activation during the transitional period of the delivery in the bloodstream cleared them from the biosystem in a faster manner; otherwise, they would have accumulated in the liver and spleen and later in other organs. 

SPIONs are known to disrupt/activate synthesis of signaling molecules, tumor antigens, formation of lysosomes, stimulate the synthesis of interleukin-8, an inflammation mediator, and disturb the cells functioning [136] The protein corona formation on graphene oxide-based nanomaterials surface is also known. The polymers coating, and conjugation have led to toxicity generation in the biosystems. The biocompatible polymers, i.e., PEG, ethylene-diamine-modified-poly-isobutylene-maleic-anhydride, polyurethane, polyethyleneimine, PPI, chondritin-4-sulphate, PAMAM and their derivatives, used in delivery of drugs [137,138,139,140,141,142] [are susceptible. In this context, it would be interesting to note that zwitterionic polymers have been reported to inhibit the formation of protein corona, at least in preliminary trials [83]. As far as graphenes are concerned, graphene oxide also produces a decrease in cell viability and induces mutagenesis. Lung injury through autophagy and chronic pulmonary fibrosis is known [143,144,145,146,147]. The involvement of liver, which sequesters 30–99% of all the administered larger sized (>100 nm) nanomaterials through the systemic circulation, lowers the nano drug delivery quotients to the intended organ. Typically, below 5% of nanomaterials, especially NPs, reach the diseased site. Kupffer and endothelial cells and hepatocytes become involved in liver damage through the elimination of Kupffer cells, increased cytokines release, tumor necrosis factor-α (TNF-α), and involvement of interleukin-1. The internal toxicity remediation involves renal, hepatic, and mononuclear phagocytic systems [148]. However, depending upon the types and composition of the nanomaterials, which includes metallic and metallic oxides nanomaterials (i.e., silver, zinc, gold, iron, manganese, cadmium, and gadolinium), the nanomaterials are variably excreted into the bile and through the bile ducts to the small intestine for excretion. Nonetheless, there are multiple iron metabolism pathways working on the SPIONs with the nanoparticles’ transformation to endogenous ferritin, which also reduce the deleterious oxidative stress [149,150,151]. The metal and metal oxides NPs with biocompatible surface chemistries, with and without biodegradable polymers have been found to be excreted out. The biodegradable nanomaterials form aggregates, reduced-sized remnants, ionic entities, and metal-protein complex for removal [152].

The polymeric nanomaterials, including synthetic and natural origins, contribute to oxidative stress, inflammation, genetic and gonadal toxicity, as well as hemo-compatibility issues, and their interactions are mediated through various biochemical routes disturbances, receptor interactions, and enzymatic activity at the polymeric nanomaterials’ interaction sites in the biosystem. Both in vivo, and in vitro toxicological evaluations against a number of cell lines, and biochemical substrates, together with relevant biomarkers have been reported [131,132,153]. The toxicity evaluations and requirements in preparative designs for nanomaterials, their size and shapes, charge, and ADME (absorption, biodistribution, metabolism, and excretion) are important factors. The nanomaterials degradation, degradants interactive potential to harm, pharmacokinetics, and toxicokinetics details to design safer nanomaterials is required. The starting springboard to formulation development is embedded in the toxicity generation understandings, whereby the safety by design (SbD), computational assessments at the molecular level toxicokinetics and its modeling, as well as methodically safe-by-design (MSbD) approaches are prime areas of focus for future studies [154,155,156]. 

## 8. Conclusions

SPIONs-based delivery has, to significant extent, allowed chemotherapeutic agents to overcome the biosystem’s barriers through site-specific magnetic targeting of the delivery. Despite significant progress, it is too early to predict viable industrial scale production of SPIONs-based delivery modules fitting a diverse range of delivery to various disease conditions that are oncological or otherwise in nature. SPIONs have the potential to operate as photothermal and photodynamic therapies, and as magnetic hyperthermia mediators. He SPIONs have been utilized to deliver cytotoxic drugs, and synthetic and biopolymeric materials, including DNA, RNAs, peptides, proteins, enzymes, antigen-antibody, aptamers, etc. into malignancies. The US FDA has approved several SPIONs based formulations (i.e., Feraheme^®^ and Feridex I.V.^®^), although none of the functionalized SPIONs have yet been approved for use in patients. Several important issues needs to be resolved at the preclinical stage, and investigations into a variety of theranostic SPIONs are required to reach the realm of clinical viability for the formulations for clinical use. The harmful side effects of other accessary components also need to be investigated for their safety and biodegradability. The practicality and consistency of mass productions of multifunctional SPIONs is another key challenge in its translation into the viable clinical format fit for patients use. Despite the fact that multiple tests have proved the safety and usefulness of employing the SPIONs, for both the diagnostic and therapeutic purposes, the commercial applications of the specialized SPIONs-based therapy modules for breast, ovarian, cervical and other cancers have remained elusive. For clinical development, and commercialization purposes, standardization, and scalable manufacturing of SPIONs have also faced several hurdles, ranging from preparation to bulk production, and the adherence and development of GMP practices, as well as imparting the specificity that is essentially required for various disease-related conditions deliveries of the nano-preparations to usher into newer nanomedicines. 

## 9. Prospects and Future Directions

SPIONs-based cancer and non-cancer chemotherapeutic agents’ deliveries hold strong promise for future development as part of personalized nanomedicine fitting the individual patients needs that have been tailor-made according to the person’s prescription. Despite the challenges, technological development for industrial-scale production of SPIONs-based specialized formulations are required. The nanomedicines promise as a first-line response drug with intelligent drug delivery and imaging agent has strong prospects for the future. Currently, no SPIONs-based drug delivery device, or similar modular options are available on the market, and the need is pressing. The difficulties are related to the mass production of appropriately designed delivery modules containing the drug(s), SPIONs, and the site-directing entities, along with the capabilities for combating other physio-chemical and biological factors for safe and effective delivery, as well as efficient live imaging and drug quantitation. These obstacles need to be overcome for a technically advanced, medically efficient, and financially viable product that can be offered to patients. Nonetheless, the SPIONs’ preparative techniques have significantly improved over the years, but specific additions are still a case-by-case requirement to technically mass produce the desired and designated SPIONs, or other metallic and non-metallic magnetization (organic magnets) entities for drug delivery and imaging. The materials and nano-preparations characterization techniques also require advancement. Although it now (ca. 2022) seems feasible to create suitable SPIONs in bulk, thereby paving the way for development of drug–SPIONs conjugates. However, the field still demands innovations and advancements in the realm of specific SPIONs-based delivery module mass productions for specific disease conditions, and also as a personalized medicine. The use of SPIONs to produce highly penetrating modules as part of transdermal patches with magnetic circuitry also need to lead to better and efficient delivery of the SPIONs carried medications at these specific locations for the efficient delivery and effective treatment that are required of any delivery precondition. SPIONs’ disposal pathways are also required to be investigated in detail for its toxicity related issues to improve the SPIONs’ desirable advantages for the targeted drug administration without any fear for safety and impending toxicity of the formulation. The combination of multiple and distinct factors of formulations for the SPIONs-based delivery module also play significant roles in the emergence of safe and effective as well as biocompatible and biodegradable SPIONs-related theranostic agent. In order to develop these types of SPIONs-based nano-systems that have optimum drug delivery capabilities, sizes, charges and loading and conjugation capabilities, need to be maneuvered through in silico approach in computer modeling, computographics, and quality by design. The chemo-biological compatibility in conjugation, overall biocompatibility of the finished product, and the total biodegradability of the SPIONs–drug conjugates are essentially of first-line of required characteristics for developing a workable module, nonetheless, needing further advancements, from the current situation, in multiple areas of cancer and other disease types, site directedness, controlled toxicity and adverse effects, as well as sterility, stability, bioavailability and biodistribution with biodegradation efficiency. The preparation, transport stability, provision of better delivery option modalities, together with delivery chronology, time-defined quantitated drug release, and the physiological and energy-need controls for the eventual delivery of the payload require thorough examination and future planning. The molecular modeling, in silico receptor-based studies on the various aspects of chemical and pharmacological characteristics of the prepared nano-conjugates also need to be understood for future development. Advancements in the sensing of drug delivery, biological safety, shelf-stability, stability during the delivery’s transitional period, and imaging of the tissues and cells at shallow and deep delivery sites are further need to be addressed through extensive investigations, as the current state of the art lacks certain parts of these requirements for safe and effective drug development, delivery, and diagnostics.

## Figures and Tables

**Table 1:** SPIONs–polymer conjugated system for DOX delivery.

Polymer	Coating/Capping	Observations	Reference
Polyethylene glycol (PEG)	Phospholipid	30.8% *w/w* loading capacity observed with optimized PEG lengths	[26]
	Silyls	Significant cytotoxicity (97.3 ± 0.8%) of SPIONs achieved	[27]
	Anti-IL4R blocking antibodies	Significant increase in cell death, apoptosis, and oxidative stress through SPIONs-IL4R-alpha and DOX	[28]
	pH-sensitive acyl hydrazone linkages	Better antitumor effect under magnetic field using SPIONs	[29]
	Bovine serum albumin (BSA)	DOX-SPIONs absorption through a combination of electrostatic and hydrophobic interactions	[30]
	Chelators and tumor-targeting ligands	cRGD-conjugated SPIONs in vitro exhibited higher levels of cellular uptake than the cRGD-free product	[31]
	Amphiphilic polymers and fluorescent dye 5-FAM	Antibody HuCC_49_DeltaCH_2_ and fluorescent dye, 5-FAM, conjugated to PEG-iron oxide NPs (IONPs)	[32]
Poly-lactic-co-glycolic acid (PLGA)	Folic acid	SPIONs reduced toxicity and improved diagnosis and treatment	[35]
	Tween 80, Brij-35, Pluronic F68, or Vitamin E-TPGS (d-α-tocopheryl polyethylene glycol 1000 succinate)	SPIONs provided efficient loading of drugs and high value of relaxivity	[37]
	Perfluorocarbon gas	In vitro delivered DOX-SPIONs microtubules enhanced ultrasound/magnetic resonance	[38]
	Per fluorooctyl bromide	High photo-thermal cytotoxicity on SKBR3 cells using SPIONs	[40]
Polyethylene glycol and poly-lactic-co-glycolic acid (PEG-PLGA)	Folate	SPIONs with synergistic cytotoxic effects in folate-overexpressing cells (HeLa and MDA-MB-231)	[42]
	D,L-Lactide and glycolide	In vitro cytotoxicity showed no cytotoxicity of DOX-SPIONs, biocompatible	[44]
	Anti-EGFR monoclonal antibody	Significant reduction in the signal intensity of A431 cells treated with SPIONs	[55]
Polyethylene glycol and 3-aminopropyl triethoxy silane (APTES)	Indium-111 (labeled) Trastuzumab	Active and passive tumor targeting by SPIONs-DOX through anti-HER2 (human epidermal growth factor receptor 2), trastuzumab antibody	[46]
	Amino acid, serine	Significant differences in amount of serine coating and enhanced drug release behavior to A549 cells	[47]
Polyethylene glycol and poly-ε-caprolactone	Disulfide bond linkage	Disulfide linkage responsible for co-polymer exhibited redox-responsive properties	[50]
DSPE-PEG 2000	Indocyanine green (ICG)	DOX-SPIONs crossed the BBB and accumulated at tumor site, seen with in vivo fluorescence and MR imaging	[52]
Polyethylene glycol and cationic polyethylene imine (PEG-PEI)	Folic acid	Increased growth inhibition with DOX-FA-SPIONs on xenograft MCF-7 breast tumor in nude mice	[54]
Poly amido amine (rPAA) with PEG-dodecyl amine	Oleic acid	rPAA-SPIONs showed better performance in mice with xenograft MDA-MB-231 breast tumor models	[57]
Poly lactic acid and poly ethylene glycol (PLA-PEG)	Folic acid	Higher cellular uptake of DOX-loaded PLA–PEG–FA-SPIONs than DOX-loaded-PLA–PEG-SPIONs	[59]
Polyethylene-oxide-trimellitic-anhydride-chloride-folate (PEO-TMA-FA)	Folate	Tumor volume decreased 4-fold with DOX-SPIONs compared to the DOXIL^®^ (commercial DOX) in rabbit models	[63]
Polyethylene imine (PEI)	Silica coat	Rapid release of DOX-SPIONs, instant tumor cell destruction	[68]
Polydopamine (PDA)	Glutathione disulfide (GS-SG)	Enhanced DOX loading efficiency at 243 mol/g achieved through SPIONs-PDA conjugation	[70]
Methoxy-PEG (mPEG)-PAMAM dendrimers	Hydrazine	Enhanced permeability of DOX to tumor by polymer-SPIONs conjugation	[80]

**Table 2:** SPIONs-conjugated systems for DOX delivery.

Polymer/Conjugating Entity	Coating/Capping	Observations	Reference
Chitosan	Poly-L-arginine-chitosan-triphosphate	Enhanced absorption of DOX at the optimum dose, validated by MRI imaging of C6 glioma cells	[110]
Dextran	β-Cyclodextrin	Optimized SPIONs formulation with a higher T(2) relaxivity, 436.8 Fe mM^−1^S^−1^	[84]
Polyaspartamide	Biotin	SPIONs enhanced the transverse 1/T2 relaxation rate for cancer detection	[92]
Graphene oxide	Gold	Improved SPIONs relaxivity	[106]
N,N-dimethyl acrylamide	N-Acryloyl glycine methyl ester	DOX-conjugated particles delivered in the U-87 glioblastoma cell line	[73]
SPIONs-hollow mesoporous silica (HmSiO_2-_SPIONs)	Fe_3_O_4_	DOX-SPIONs through HmSiO_2_ conjugation integrated into HeLa cells	[77]
2-(2-Methoxy)ethyl methacrylate (MEO_2_MA)	Oligo(ethylene glycol) methacrylate (OEGMA)	DOX-SPIONs for cytotoxicity against SKOV-3 cells in human ovarian cancer	[78]

**Table 3:** Different parameters of the polymeric SPIONs conjugates in the delivery of DOX.

Polymer Conjugate/Modification/Coating	Size (nm)	Zeta Potential(mV)	Drug Loading (%)	Drug Release (h/%)	Reference
PEG 2000-DSPE	22.9	−38.19	4.86%	37% (10 h)	[52]
DSPE-2000-phospholipid	14	-	30.8%, *w/w*	20% (2 h)	[26]
PEGylated	44.6 ± 20.3	−26.1	6.91± 0.47%	33.4–42.0% (24 h)	[32]
PEGylated-Silica	20 ± 3	−2.27	69.3 ± 1.4%	23 ± 2.2% (24 h)	[27]
PLA-PEG	41.98 ± 3	−25.6	79.6 ± 6.4%	92% (2 h)	[59]
PLGA-perfluorooctyl bromide	296	-	39 ± 1.45%	100% (52 h)	[40]
PLGA-FA	85	-	-	63.3%	[35]
PLGA-PEG-folate	170	~-33	-	~4.47 folds	[42]
PLGA:PEG(4000)	30–60	−17.4	78%	30.1% (12 h)	[44]
NIPAM-MAm-HmSiO_2_	100–300 nm	−19.0	95%	-	[77]
GO-APTES	260	−3.18 ± 1.07	61.42%	-	[105]
pDA-GSSG	33 ± 5	−26.1	78%	-	[70]
TMSP-EDT	51.8 ± 1.3	−27.3 ± 1.0	5 ± 0.05%	42% (3 h)	[103]
Spider silk	112	−25.3 ± 0.9	-	80% (7 d)	[97]
Chondritin-4-sulfate (CS)	91.2	−49.1 ± 1.66	-	96.67% (24 h)	[88]
CM-ABRS	205.6 ± 0.211	−26.1	6% (*w/w*)	68.23% (24 h)	[116]

**Table 4:** Different parameters of the SPIONs–polymer conjugates for the delivery of DOX.

Polymer Conjugate (Modified/Coated)	Cytotoxicity (Cells Viability, %)	IC_50_	Cell Line	Reference
PEG-PAMAM	28–50%	32 µM	Caco-2	[56]
DSPE-PEG 2000	90%	100 µg/mL	U251	[52]
DSPE-2000-phospholipid	64 ± 11%	2.42 μg/ml	HeLa	[26]
PEGylated-silica	88 ± 5.2%	500 μg/mL	L929	[27]
PEG and PEI-FA	60%	4 μg/mL	MCF-7	[54]
PLA-PEG-FA	82.83 ± 4.1%	5 mg/mL	HeLa	[59]
PLGA-FA	60%	0.132 μM	A549	[35]
Chondritin-4-sulfate	98%	7.1 mm	A2780	[86]
GO-APTES	37%	~0.3 μg/mL	HeLa	[105]
GO-β-CD	50%	1 μM	MCF-7	[104]
pDA-GSSG	37.1 ± 9.5%	334.4–836.1 nM	PC3	[70]
TMSP-EDT	40%	300 ng/mL	U251	[103]
CM-ABRS	50%	5.9 ± 0.13 μg/mL	HeLa	[116]

## Data Availability

Not applicable.
